# Strategies to measure and improve emergency department performance: a scoping review

**DOI:** 10.1186/s13049-020-00749-2

**Published:** 2020-06-15

**Authors:** Elizabeth E. Austin, Brette Blakely, Catalin Tufanaru, Amanda Selwood, Jeffrey Braithwaite, Robyn Clay-Williams

**Affiliations:** Australian Institute of Health Innovation, Macquarie University, Sydney, Australia

**Keywords:** Length of stay, Observation units, Patient satisfaction, Point-of-care testing, Process re-design, Wait-time

## Abstract

**Background:**

Over the last two decades, Emergency Department (ED) crowding has become an increasingly common occurrence worldwide. Crowding is a complex and challenging issue that affects EDs’ capacity to provide safe, timely and quality care. This review aims to map the research evidence provided by reviews to improve ED performance.

**Methods and findings:**

We performed a scoping review, searching Cochrane Database of Systematic Reviews, Scopus, EMBASE, CINAHL and PubMed (from inception to July 9, 2019; prospectively registered in Open Science Framework https://osf.io/gkq4t/). Eligibility criteria were: (1) review of primary research studies, published in English; (2) discusses a) how performance is measured in the ED, b) interventions used to improve ED performance and their characteristics, c) the role(s) of patients in improving ED performance, and d) the outcomes attributed to interventions used to improve ED performance; (3) focuses on a hospital ED context in any country or healthcare system. Pairs of reviewers independently screened studies’ titles, abstracts, and full-texts for inclusion according to pre-established criteria. Discrepancies were resolved via discussion. Independent reviewers extracted data using a tool specifically designed for the review. Pairs of independent reviewers explored the quality of included reviews using the Risk of Bias in Systematic Reviews tool. Narrative synthesis was performed on the 77 included reviews. Three reviews identified 202 individual indicators of ED performance. Seventy-four reviews reported 38 different interventions to improve ED performance: 27 interventions describing changes to practice and process (e.g., triage, care transitions, technology), and a further nine interventions describing changes to team composition (e.g., advanced nursing roles, scribes, pharmacy). Two reviews reported on two interventions addressing the role of patients in ED performance, supporting patients’ decisions and providing education. The outcomes attributed to interventions used to improve ED performance were categorised into five key domains: time, proportion, process, cost, and clinical outcomes. Few interventions reported outcomes across all five outcome domains.

**Conclusions:**

ED performance measurement is complex, involving automated information technology mechanisms and manual data collection, reflecting the multifaceted nature of ED care. Interventions to improve ED performance address a broad range of ED processes and disciplines.

## Introduction

Over the last two decades, Emergency Department (ED) crowding has become an increasingly common occurrence worldwide [1]. EDs must continue to provide care during periods of crowding, and respond to expected changes (e.g., seasonal increase in demand) and unexpected changes (e.g., unanticipated events and varying demand) [2]. However, crowding impedes ED staffs’ capacity to provide timely, safe and quality care. It extends the time patients spend in ED, and threatens patient outcomes [3].

Crowding in EDs is the product of input, throughput and output factors such as the volume of patients arriving to be seen, the time taken to assess and treat patients, and the availability of beds in hospital wards [4]. Interventions (e.g., decision-making structure, resource allocation, procedures) to address these factors have been widely implemented, with mixed results [5–8]. Identifying effective interventions known to have improved care can support the uptake of those interventions in different contexts. Understanding the characteristics of those interventions and their limitations can inform the development of new strategies to address common patient flow problems.

Ideally, the design and selection of performance measures should align with the system’s purpose and improvement strategy in order to identify the extent to which the system is working effectively. It is unsurprising then that input, throughput and output measures such as wait-time, length of stay and patient satisfaction have been used to report on EDs’ performance [4, 9]. Understanding how ED performance has been measured in the past will support the selection of measures and inform the development of new measures to address gaps in performance knowledge.

The purpose of this scoping review was to map the research evidence provided by reviews on strategies to measure and improve ED performance. The review questions addressed were: (1) how is ED performance measured, (2) what are the interventions used to improve ED performance and (3) what is the role(s) of patients in improving ED performance, and (4) what are the outcomes attributed to interventions used to improve ED performance.

## Methods

### Study design

We conducted a scoping review of the literature from inception of bibliographic databases to July 2019 related to strategies to measure and improve ED performance. The study protocol was prospectively registered in December 2018 in the Open Science register (https://osf.io/73r4t). This protocol guided the review in adherence with the preferred reporting items for systematic reviews and meta-analyses statement (PRISMA) [10].

### Inclusion criteria

Systematic reviews of primary research studies, reviews of reviews (umbrella reviews), and other research syntheses not fulfilling all criteria for systematic reviews published in the English-language peer-reviewed literature were included that met the following additional criteria: (1) review studies involving clinicians, patients, and/ or administrators in the ED or review studies that measure ED performance without involving participants (e.g., Length of Stay or patient mortality retrieved from aggregate hospital data); (2) discusses a) how performance is measured in the ED, b) interventions used to improve ED performance and their characteristics, c) the role(s) of patients in improving ED performance, and d) the outcomes attributed to interventions used to improve ED performance; (3) focuses on studies in a hospital ED context in any country or healthcare system.

### Search strategy

To identify eligible studies, we developed a comprehensive search strategy using medical subject headings and text words for the general concepts of performance measures, interventions, and patient involvement. Cochrane Database of Systematic Reviews, Scopus, Embase, CINAHL and PubMed were searched on 14 January 2019. No date limits were used. English only publications were considered. An updated search was completed on 9 July 2019 and included a date filter (publications from 1 January 2019 to 31 December 2019). The full search strategy for all databases is shown in Appendix A (See Additional file 1).

An example, illustrating the search strategy for PubMed, is as follows:

((((emergency Service, Hospital [mh]) OR emergency department [tw])) AND (((((quality of health care [mh]) OR quality improvement [mh]) OR quality [tw]) OR improvement [tw]) OR performance [tw])) AND ((((((((review [ti]) OR systematic review [ti]) OR meta-analysis [ti]) OR meta-synthesis [ti]) OR scoping review [ti]) OR integrative review [ti]) OR overview [ti]) OR umbrella review [ti])

### Study selection

The results of the searches were entered into EndNote citation management software (version 8.2; Thompson Reuters, New York, NY), and duplicates were removed. For each review, title, abstract, and full-text were independently screened by pairs of reviewers for inclusion according to pre-established criteria. Disagreements were resolved via discussion. Abstracts flagged as potentially relevant by reviewers underwent full-text review.

### Data extraction and quality assessment

The data was extracted by independent reviewers by using an extraction tool specifically designed for the review. The data extraction form was piloted for usability prior to data extraction. The extraction form included information on Author(s), year of publication, country where review was conducted, type of review, review objectives and questions, number of studies included, types of intervention/s, intervention characteristics, type of measure used and/ or type of outcome measured.

The quality of the included papers was assessed using the Risk of Bias in Systematic Reviews (ROBIS) tool for assessing the risk of bias in systematic reviews [11]. The purpose of this assessment was only to allow for the quality of the included reviews to be mapped/ described. Prior to critical appraisal, the ROBIS was piloted on a sample of reviews. The quality of included reviews was explored by pairs of independent reviewers. Disagreements were resolved via discussion.

### Data processing and analysis

A narrative synthesis was performed for this review, including numerical statistical summaries, textual commentaries, and tabular and graphical representations.

## Results

**Fig. 1:**
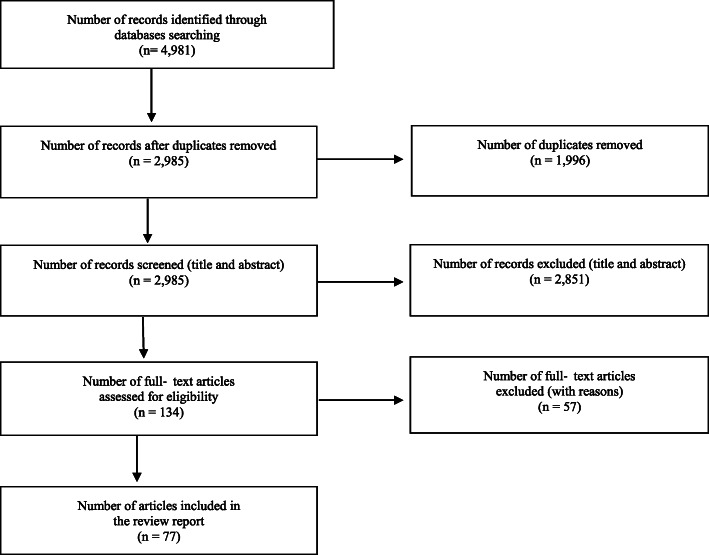
PRISMA flow diagram for study selection

The combined searches yielded 4981 articles, including 1996 duplicate articles. Of these, 2985 abstracts and 134 full-texts were reviewed with 77 articles meeting inclusion criteria. Figure 1 illustrates the PRISMA diagram for the identification, screening, and inclusion processes.

**Fig. 2:**
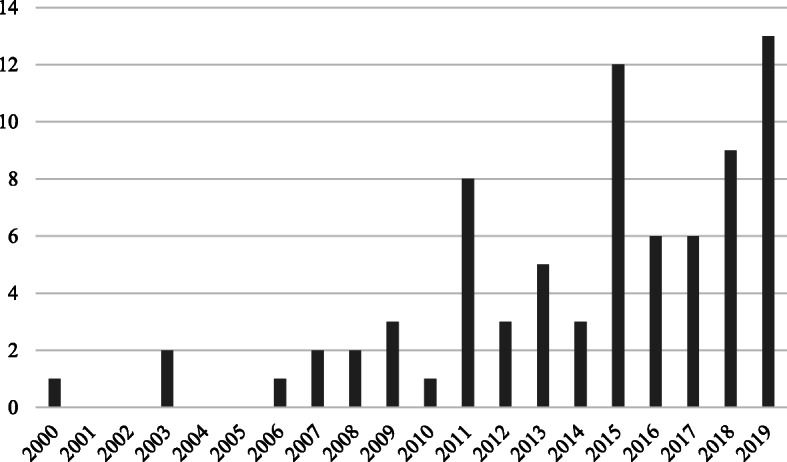
Distribution of published reviews over time

**Fig. 3:**
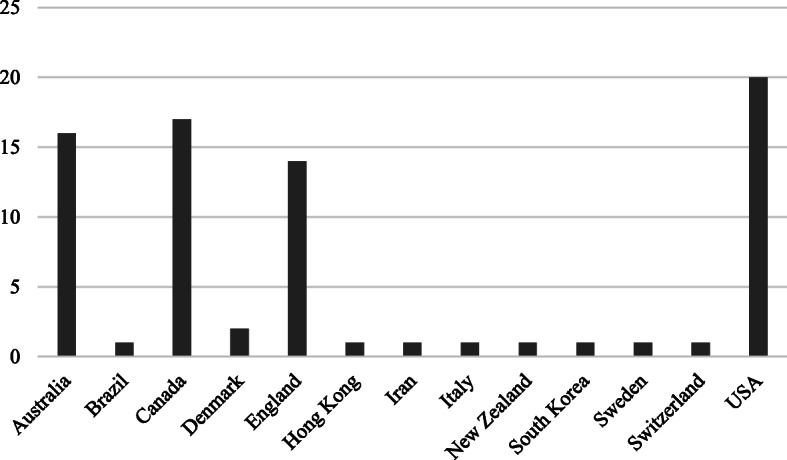
Distribution of published reviews by country affiliation of first author

An additional table outlines the characteristics of the included articles addressing ED performance measures (See Additional file 2). An additional table outlines the characteristics of the included articles addressing interventions, and patient role in ED performance (See Additional file 3). An additional table outlines the evidence contribution of the included reviews to each review question (See Additional file 4). Distribution of included reviews published per year (2000–2019) is provided in Fig. 2. Figure 3 shows the distribution of locations where published reviews were conducted (based on the country affiliation of the first author).

### Quality assessment

**Fig. 4:**
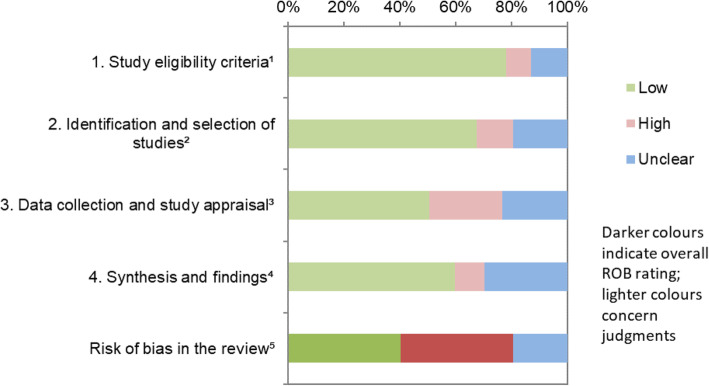
Graphical presentation for ROBIS results across all included reviews. ^1^ study eligibility criteria prespecified and appropriate for the review question. ^2^ sensitivity of the search. ^3^ rigour of the data collection process. ^4^ appropriateness of the synthesis for the review question. ^5^ overall risk of bias. “Low”, “high”, or “unclear” represents the rated level of concern about bias associated with each domain [11]

Using the ROBIS tool, 31 reviews were assessed as being low bias, 31 as high bias, and 15 as unclear bias. The ROBIS risk of bias assessment results for each domain and the overall risk of bias for individual reviews are presented in an additional file (See Additional file 5). Figure 4 displays a summary ROBIS assessment across all included reviews, graphically presenting the results of the ROBIS assessment for each domain and the overall rating.

### How ED performance is measured

Three reviews reported on ED performance measures [12–14]. Madsen (2015) extracted evidence for 202 individual indicators of ED performance from 127 articles, categorising them into process (e.g., time to diagnosis, physician workload), outcome (e.g., mortality, ambulance diversion), satisfaction (e.g., rate of complaints, patient participation in own care), structural/ organisational (e.g., admission rate, resources) and equity (e.g., weekday/weekend variation, sex, race, age) performance measures. Data sources used to generate the indicators are ED information technology, questionnaires, chart reviews, and multihospital databases [12]. Sørup (2013) identified 55 different performance measures and categorised ED performance measures into patient related measures focusing on safety (e.g., medication errors, unplanned reattendance), patient centeredness (e.g., complaints, left-without-being-seen), and satisfaction; employee related measures focusing on occupational profile (e.g., educational positions), and work environment; and operational performance focusing on planning (e.g., occupancy rate), utilisation (e.g., number of ECG’s taken, number of consultations), efficiency (e.g., ED admission transfer rate, length of stay), and time intervals (e.g., time to registration, triage, treatment). Stang (2015) examined crowding measures linked with quality of care including ED volume, number of patients in the waiting room, and ED length of stay.

### Interventions used to improve ED performance and their characteristics

The remaining 74 articles addressed interventions used to improve ED performance. Interventions to improve ED performance address either practices and processes or team composition. Interventions addressing how tasks were performed in ED were identified as practice and process interventions. Interventions addressing the discipline or training of professionals practicing in ED were identified as team composition interventions.

### Practices and processes

Six domains of clinical practice and processes have been targeted for interventions. They are: triage, care transitions, process re-design, point-of-care testing, observation units, and technology.

#### Triage

Designed to expedite care, triage processes sort patients according to urgency or type of service required [15]. Twelve reviews examined interventions relating to triage systems and processes. Triage based interventions included having a physician present [8, 16–19] also called a triage liaison physician [20], a triage team consisting of at least 2 medical personnel (nurse or physician) [21, 22], dedicated triage resources (e.g., ECG machine and ECG technician [23]), triage education [23], variations of basic triage [15], triage protocols [24, 25], and nurse-led triage services [26]. A description of the characteristics for each type of triage intervention is provided in Table 1.

**Table 1:** Triage interventions and intervention characteristics

Intervention	Intervention Characteristics
**Senior Doctor Triage/ team triage**	Designed to allow for rapid medical intervention and care escalation, senior doctor triage/ team triage involves the presence of a (senior) emergency doctor (physician) in triage to identify potential emergencies, initiate diagnostics and treatment prior to patients being seen in ED [8, 15–22].
**Dedicated Triage Resources**	Dedicated triage resources include a dedicated ECG technician and machine, and the creation of a dedicated ECG room with two stretchers beside triage [23]
**Triage Education**	Staff education about atypical presentations, signs and symptoms, as well as how to perform ECGs have been implemented [23]
**Triage Systems**	Variations in triage systems include prioritising patients without providing treatment, prioritising patients while providing simple treatment and formal triage systems [15]
**Triage Protocols**	Triage protocols procedures for specific symptoms and treatments, for example, nurse-requested radiograph protocol outlines the rules and procedures under which nurses can/ should request radiographs for patients at triage [24]
**Nurse-led Triage**	Nurse-led-triage involves triage by a Nurse Practitioner, Advanced Nurse Practitioner, Emergency nurse Practitioner, or ED nurse [26]

#### Care transitions (handover processes)

Care transitions involved patient handover, which is the process of transferring accountability and responsibility for patient care to another person [27]. Seven reviews examined interventions relating to patient handover processes and transitions in care. Patient handover processes and transitions in care interventions include handover tools [27–30], bedside registration [19, 31], discharge planning [31], discharge communication [32], process protocols and guidelines [27, 29, 30], handover training [29], dedicated offload nurse for triaging and assessing EMS patients [30], nurse discharge coordinators [33]. A description of the characteristics for each type of care transition intervention is provided in Table 2.

**Table 2:** Care transition interventions and intervention characteristics

Intervention	Intervention Characteristics
**Handover Tools**	Handover tools standardise communication using a structured information template performed either verbally or nonverbally [28]. For example, Identification, Situation, Background, Assessment, Requirements and Requests (ISBAR) is a tool for face-to-face beside handover [27–30]. Other tools include Situation, Background, Assessment, Responsibilities and Risks, Discussion and Disposition, Read-back and Record (SBAR-DR) model for verbal handoff, Mechanism of Injury/Illness, Injuries, Signs, observations and monitoring, and Treatment given (MIST; DeMIST includes Demographic information to the handover), hospital developed handover tools, as well as written handover, handover added to patients paper chart, and an eSignout step added to the ED dashboard [27–30].
**Bedside Registration**	Bedside registration immediately following tirage involves, when beds are available, patients are brought immediately through to the patient care area following triage where they are registered by a clerk whilst simultaneously being assessed by medical staff [19, 31].
**Discharge Planning**	At a clinical level, discharge planning involves the early planning of patients’ care after discharge [31]
**Discharge Communication**	Discharge communication should include important information about the illness, verification of comprehension, and tailoring discharge instructions to address areas of misunderstanding [32]. Interventions to improve discharge communication include education or the sharing of information with patients and the different modes through which information is delivered (e.g., video, interactive websites, written, face-to-face), overcoming existing barriers, or providing additional support to encourage a specific behaviour [32].
**Discharge Protocols**	Closely linked with handover tools, process protocols and guidelines outline procedures and rules clarifying the transfer of responsibility, as well as a structure for handover [27, 29, 30]. Handover training lasting 3 h, covered five rules of communication, case scenario simulation and a handover protocol [29]
**Discharge Clinical Roles**	A new clinical role in the form of a dedicated offload nurse for triaging and assessing EMS patients [30]. Another role developed to facilitate handover is the nurse discharge coordinator [33]. The nurse discharge coordinator intervention involves the role discussing with the patient, their health care needs, education, referral to outpatient facility, 24 h nurse follow-up, back-up consultation 1 week after discharge [33]

#### Process re-design

Process re-design refers to changes in how tasks are performed. Twenty-three reviews examined interventions relating to process re-design. Processes that have been re-designed included clinical guidelines and protocols [25, 31, 34–36], patient assignment and referral processes [4, 31, 36–38], organisational processes (e.g., communication, administration) [19, 25, 35, 39, 40], nurse-initiated care processes [8, 17, 21, 41–44], clinical decision supports [45–47], and lean management/ lean thinking interventions [48–50]. A description of the characteristics for each type of process re-design intervention is provided in Table 3.

**Table 3:** Process re-design interventions and intervention characteristics

Intervention	Intervention Characteristics
**Guidelines and Protocols**	Process redesign also refers to changes to existing or the introduction of evidence-based clinical practice guidelines for specific conditions [31], protocols (pathology: [31]; treatment: [25]; medication: [34, 42], mandating the redundant reading of emergency CT scans [35].
**Patient Assignment and Referral Processes**	Patient assignment processes [31], and referral processes [4, 31, 36]. Case management involves the identification of appropriate providers and services for individual patients based on a continuous, integrated medical and psychosocial model of care [37, 38].
**Organisational Processes**	Communication and consultation intervention between radiologists and ED physicians to reduce patient call backs to the ED [35], administrative interventions (e.g., outsourcing environmental services, [25]), the addition of administrative, clinical and ancillary personnel [39], logistical changes in radiology and laboratory [39], rearranging bed zones [39], performance targets (e.g., the 4 h rule, disposition [19, 40];.
**Nurse-Initiated Care Processes**	Nurse-initiated care processes consist of various interventions relating to nursing activities [8]*.* Reviews examined interventions relating to nurse-initiated care processes in ED including medication [41, 42], x-ray [8, 17, 21, 43, 44], protocols [19], and diagnostic tests [44].
**Clinical Decision Supports**	Clinical decision supports refers to the use of a validated clinical decision rule to assess the pre-test probability of the diagnosis [45, 46] or tool to assess the need for diagnostic investigations [47]. For example, tools to assess the need for imaging in adult patients include the National X-radiography Utilization Study (NEXUS) criteria, and the Canadian C-Spine Rule (CCR [47];).
**Lean Management/ Thinking**	Lean management/ thinking is a suite of concepts, methods and tools developed by Toyota Motor Corporation [48–50]. Lean processes are designed to improve productive capacity and reduce waste [48–50]. Three reviews examined Lean interventions in ED [48–50]. Lean has been applied in a number of ways. For example, designing a detailed map of the process (Value Stream Map – VSM) to identify waste and bottlenecks [48], streaming patients according to severity, dedicating different ED spaces for different types of patients [48] with dedicated nurse and physician for the different areas [48]. Other lean interventions included computer systems implantation, changes in roles and responsibilities, flow managers and screening nurses [48]. Kaizen events moderated by lean consultants or lean specialist (VSM, leadership involvement, boot camp, reallocation of staff, commitment of the department chairperson, communication board, periodic electronic communication [48];). Process changes such as new processes and related operating procedures including eliminating outdated policies, fast-track process for low complexity patients. System changes include data collection and monitoring (e.g., weekly review, quality improvement measurements taken and shared with staff), education/ training (orientation to the new process, posting process map in public areas), tools/ technology (standardised forms, checklists), communication and teamwork (communication tools, team assessment of patient history), staffing reassignment/ new roles/ responsibilities (reassignment to match peak patient volume or arrival rates, dedicated ECG and laboratory technician in ED, reassignment/ reorganisation of space (e.g., space reallocated for rapid assessment and holding patients, designated physician examination rooms), other changes (stocking done as needed, improved signage, celebrating goal achievements) [49]. Lean intervention team composition included, hospital management team or the head of ED, physicians, nurses, staffs and external counsellors, as well as external consultants (experts in lean [50];.

#### Point-of-care testing

Point-of-care testing refers to laboratory analysis located in the ED [8, 21]. Five reviews examined point of care testing in ED [8, 19, 21, 51, 52]. Point-of-care testing has been used for a range of diagnostic tests including cardiac troponin [51], metabolic [19], urinalysis, pregnancy testing, cardiac markers, glucose [19], influenza, and respiratory syncytial virus [52].

#### Observation units

Observation Unit interventions refer to ED-based observation units [31]. Twelve reviews examined observation units in ED [4, 8, 17–19, 25, 31, 39, 53–56]. ED based observation units have been developed for specific clinical needs such as Chest pain and Asthma [31, 39], for specific processes such as assessment and procedures (e.g., Rapid Assessment Zones/ Pods) [18, 55], medically stable patients likely to require admission (e.g., Medical Assessment Units) [4, 8, 17], or further investigations (e.g., Short Stay Units) [8, 17, 54, 56], management for more than 4 h (e.g., ED managed Acute Care Unit) [19], or to manage referrals from GPs (e.g., Quick Diagnostic Units) [18]. A description of the characteristics for each type of process re-design intervention is provided in Table 4.

**Table 4:** Observation Unit interventions and intervention characteristics

Intervention	Intervention Characteristics
**Condition Specific Observation Units**	For example, Chest Pain Observation Units are for patients presenting with chest pain who are a low risk of acute myocardial infarction to undergo a short period of monitoring with serial ECGs and cardiac enzymes before further testing and discharge [53].
**Rapid Assessment Zones/ Pods**	Rapid Assessment Zones/ Pods (also referred to as Minor Injury Units) are spaces in ED adapted for clinician assessment and procedures for patients whose therapeutic needs exceed typical fast-track criteria, but can still receive investigations/ therapy in a chair and require limited observation [18, 55]. In Rapid Assessment zones/ pods, investigations are initiated, patients wait for results and/ or receive treatment in a chair or stretcher [55].
**Medical Assessment Units**	Medical Assessment Units are areas in ED for patients with complex medical conditions who will likely require admission [4, 8, 17]. Medical Assessment Units involve fast-tracking care of medically stable patients [17].
**Short Stay Units**	Short Stay Units are spaces in ED for patients who require a short period of observation, treatment (e.g., blood transfusions), or further diagnostic investigations that may take several hours to resolve without occupying ED beds or being admitted [8, 17, 54, 56].
**ED Managed Acute Care Unit**	ED managed acute care unit is a space physically remote from ED but staffed by ED for ED patients who require observation or management for more than 4 h [19].
**Quick Diagnostic Units**	Quick Diagnostic Units have been introduced to ED to manage referrals from GPs to EDs and are staffed by internal medicine specialists [18]. ED-based observations similar to the Quick Diagnostic Unit include Clinical Decision Units, Medical Assessment and Planning Units, Rapid Assessment and Planning Units, Observation bays, Express Admission Units [18].

#### Technology

Technology has been increasingly integrated into the ED [57]. Seven reviews examined interventions addressing technology in the ED. Technology has been introduced into EDs in the form of health information technology such as computerised clinical support systems (e.g., decision supports and provider entry forms) [45, 58], mobile devices [57], and telecommunication technology [59], computer simulation [60], and eHealth records access [61, 62]. A description of the characteristics for each type of technology intervention is provided in Table 5.

**Table 5:** Technology interventions and intervention characteristics

Intervention	Intervention Characteristics
**Computerised Clinical Support Systems**	Computerised physician order entry [45] and computerised provider entry forms provide clinicians with timely electronic access to patient information and electronic decision support (e.g., alerts, reminders, order sets [58];).
**Mobile Devices**	Different types of mobile devices/ workstations have been employed in ED including hand held personal digital assistant, wireless computers/ mobile work stations, iPod® device [57].
**Telecommunication Technology**	Telecommunication technology (e.g., transmission of video, images, radiological studies, physiological data, and pathology results) to provide care to a patient typically distal from the provider [59].
**Computer Simulation**	Computer simulation and modelling interventions use simplified representations of reality to analyse ED patient flow and resource capacity planning [60].
**eHealth Records Access**	Electronic health records use health information technology to allow virtual health information management and exchange [62]. Two reviews examined eHealth records access in ED [61, 62]. Shared electronic health records (e.g., summary of care records, virtual health record) involved making patient care records (e.g., GP records) available to providers of emergency care [61]. Health information exchange programs can include the sharing of laboratory and imaging tests associated with episodes of care [62].

### Team composition interventions

Different roles and specialties have been integrated into the ED. These included advanced nursing roles, physiotherapy, general practitioners, scribes and physician assistants, pharmacy, and mental health services, as well as the development of professional skills.

*Advanced Nursing Roles*. Seven reviews examined interventions relating to advancing nursing roles in the ED. Advanced nursing interventions primarily include the nurse practitioner role [17, 18, 63–66] sometimes called advanced nurse practitioner/ advanced clinical practitioner/ advanced practice nurse [66, 67], clinical nurse specialists [65], certified registered nurse anaesthetists [65], and Clinical Initiatives Nurse (CIN [17, 68];. Advanced nursing roles typically require further education and require a minimum of 2 years emergency nursing experience [68]. A description of the characteristics for each type of advanced nursing role intervention is provided in Table 6.

**Table 6:** Advanced Nursing Role interventions and intervention characteristics

Intervention	Intervention Characteristics
**Nurse Practitioner**	An ED Nurse practitioner in an independent practitioner whose knowledge and skills allow them to make assess, diagnose, treat, prescribe and refer patients to other health specialties [17, 63, 64]. Nurse practitioners may be required to be covered by their own malpractice insurance and own license [63]. Nurse practitioner practice, and therefore interventions, vary considerably [18]. Nurse practitioners generally manage patients presenting with minor injuries or illnesses [18, 67].
**Clinical Nurse Specialist**	Clinical Nurse Specialists are midlevel practitioners who are certified in a speciality [65]
**Certified Registered Nurse Anaesthetists**	Certified Registered Nurse Anaesthetists are midlevel practitioners with qualification and accreditation to administer anaesthesia [65]
**Clinical Initiatives Nurse**	Clinical Initiative Nursing roles provide as early as possible, assessment, initiation of diagnostics, and implementation of management strategies for patients with a range of conditions in ED waiting rooms, prior to being seen by a medical officer [68]. The CIN role in ED supports triage nurses and utilises advanced nursing practices such as nurse-initiated activities (e.g., analgesia, and x-rays [17];).

#### Physiotherapy

Three reviews examined interventions relating to physiotherapy roles in ED [69–71]. The role of physiotherapists in ED includes the assessment and management of acute and subacute musculoskeletal conditions, recent burns and diabetic wounds, provision of in-service training to other ED staff, liaising with nursing, medical, and allied health staff, and ensuring safe discharge from ED including arranging community services [69–71]. Physiotherapists have also been trained to read and request imaging and to prescribe a limited number of medications [69, 70].

#### General practitioners

Two reviews examined interventions relating to general practitioner roles in ED [72, 73]. There are different models in which general practitioners have been introduced into ED [72, 73]. General practitioners have been used to staff non-urgent (rather than urgent) streams when patients are triaged into separate streams [72, 73]. General practitioner services are also available onsite next to the ED and patients self-select or are redirected to these services from the ED. General practitioners have also been involved in the triage of patients presenting to the ED [72, 73]. General practitioners have also been fully integrated into ED, providing care jointly with ED staff on a range of primary care and higher acuity emergency cases [72, 73].

#### Scribes and physician assistants

Four reviews examined interventions relating to models of care using support staff such as scribes and physician assistant roles in ED [8, 18, 74–76]. A description of the characteristics for scribes and physician assistants interventions is provided in Table 7.

**Table 7:** Scribe and Physician Assistant interventions and intervention characteristics

Intervention	Intervention Characteristics
**Scribe**	Scribes are non-licensed health care team members that follow ED doctors during patient care to concurrently document patient history, physical examination, and procedures in an accurate manner as it is being done by the ED doctor [74, 76]. Scribes keep track of laboratory findings and radiological studies, prompt doctors to review test results, assist with referrals, and record other pertinent information [74, 76].
**Physician Assistant**	Physician Assistants are fully licensed medical practitioners who are trained to provide care under the direction and supervision of a doctor [75]. While the doctor is ultimately responsible for the patient and established the degree of supervision, physician assistants have autonomy in medical decision making [75]. Typical duties include history taking, physical examination, evaluating laboratory data, instituting treatment, performing procedures screening ED patients with routine problems, admitting certain patients and communicating with consultant services [75].

#### Pharmacy

Two reviews examined interventions relating to pharmacy roles in ED [77, 78]. The scope of pharmacy roles in the ED varied. In the ED, pharmacists conduct consultations including interpreting results and providing pharmacotherapy recommendations [77, 78]. ED pharmacy programs also included pharmacists tracking patients medication due times for repeat medications, completing medication histories, documenting patient body weight, height, and allergies [77, 78]. Pharmacists have also been involved ED patient follow-up on culture and susceptibility results, adjusting or discontinuing therapy as needed [77, 78].

#### Mental health services

Two reviews examined interventions relating to mental health services in ED [79, 80] including Liaison Mental Health Services [79, 80], co-located Psychiatry Liaison Personnel/ Spaces [80], Psychiatry Specialist Services [80]. A description of the characteristics for each type of mental health services intervention is provided in Table 8.

**Table 8:** Mental Health Services interventions and intervention characteristics

Intervention	Intervention Characteristics
**Liaison Mental Health Services**	Liaison mental health services have been located in general hospitals outside of ED, but also located inside EDs [79]. Liaison team composition varies and can include nurses, social workers, psychologists, and psychiatrists [79]. Liaison mental health services see clients directly (most referrals involve 60 min of contact with clients) in both initial and follow-up face to face contact with clients [79]. Liaison mental health services also perform administrative, supervision, audit and research, teaching, and meetings [79]. Some models of liaison mental health services include the integration of extra specialist mental health staff (mental health nurses rather than upskilled ED trained staff) as part of the full time ED team and involved in patient triage, mental health patient assessment, management, referral and liaison with other services [80].
**Co-located Psychiatry Liaison Personnel/ Spaces**	co-located psychiatry liaison personnel or spaces for patients [80] are not integrated into the normal ED team, but could be called upon to see mental health patients in the ED or in a bespoke space [80].
**Psychiatry Specialist Services**	Psychiatry specialist services review and care for ED mental health patients [80]. These teams include social workers, psychiatrist and psychologists who come to the ED after referral from the ED staff [80]. Daily rounds by a psychiatrist in the ED has also been implemented [80].

#### Professional development

Nine reviews examined professional development interventions in ED. Professional development interventions included eight-hour customer service training related to applying industry customer service principles to health care, benchmarks, and taught customer service skills such as negotiating agreement and resolution of expectations [31, 39]; and a 10 week medical Spanish language course [39]. The provision of audit/ feedback (from a supervisor/ colleague/ external coder) on clinical practice has been implemented in a variety of formats including weekly case specific, every 6 weeks individual feedback with group discussion; or individual feedback provided via email, written, verbal, electronic, and combination of media, one on one, group, (e.g., patient outcomes, quality of documentation [81–83]. Other interventions include cross-training nurses to care for patients in a designated area [25], monthly staff education/ workshops about hand hygiene with elements of targeted feedback [84], and clinical education to improve nurses’ and medical staffs’ knowledge of pain management through an education program [42, 85].

### The role(s) of patients in improving ED performance

Patients are consumers of healthcare services provided by EDs. The delivery of healthcare depends on the relationship between clinicians and patients and the degree to which patients play an active or passive role [86]. Two reviews examined the role of patients in improving ED performance [42, 86]. Patients’ role in improving ED performance has been primarily addressed by involving patients in shared decision making. Shared decision-making involves active patient involvement with the clinician, sharing information and collaboratively taking steps to reach agreement about which treatment to implement [86]. Shared decision making has been addressed through decision supports [86], and education [42]. A description of the characteristics for each type of patient role intervention is provided in Table 9.

**Table 9:** Patient Role interventions and intervention characteristics

Intervention	Intervention Characteristics
Decision Supports	Decision support interventions are designed to support patient involvement in decisions about care for bactremia and associated complications in febrile children, laceration repair in children, rehydration options, and risk of acute coronary syndrome [86]. Paper based decision support interventions convey aggregate level information on risks and benefits of treatment options [86]. The use of computerised methods to generate outcome probabilities for individual patients using embedded statistical models [86].
Education	Parental/ family education has also been implemented through a pain management booklet and bookmark, a ‘pain passport’ which actively engaged parents and children in pain management discussions with nurses encouraging children and parents to monitor and track the child’s pain score during their ED stay [37].

### The outcomes attributed to interventions used to improve ED performance

The outcomes attributed to interventions used to improve ED performance identified by the review can be categorised into five key areas: Time, proportion, process, cost, and clinical outcomes. Time-based measures record time stamps/ intervals, and sub-cycle intervals [25]. Measures of time intervals varied, however, the most commonly used were length of stay (LOS) in ED and waiting time. Proportion-based measures record elements of ED performance rates [25]. Measures of proportion-based measures varied widely and included admissions, resource use, and treatment follow-up rate. Process-based measures document elements of ED process performance [25]. Direct and indirect measures of quality of care, including left without being seen, did not wait, as well as patient and provider satisfaction, were commonly reported ED process performance measures. Cost-based measures indicate the financial implications of health care provided. Measures of cost varied and lacked detail, and were often reported simply as “costs” [18, 51, 62]. Clinical-based measures indicate the medical outcomes for patients of the health care provided. Measures of clinical outcomes reported varied, however, and the most commonly used were adverse events and readmission.

#### Practices and processes

Overall, time-based and process-based outcome domains were the most widely used measures for interventions with 24 out of the 30 individual interventions reporting at least one of each of these domains. Proportion-based outcomes were similarly well reported on for interventions with 21 out of the 30 interventions reporting proportion-based measures. Cost-based and clinical-based outcomes were the least utilised domains with only 12 and 17 interventions respectively reporting at least one outcome in these domains.

#### Team composition

**Fig. 5:**
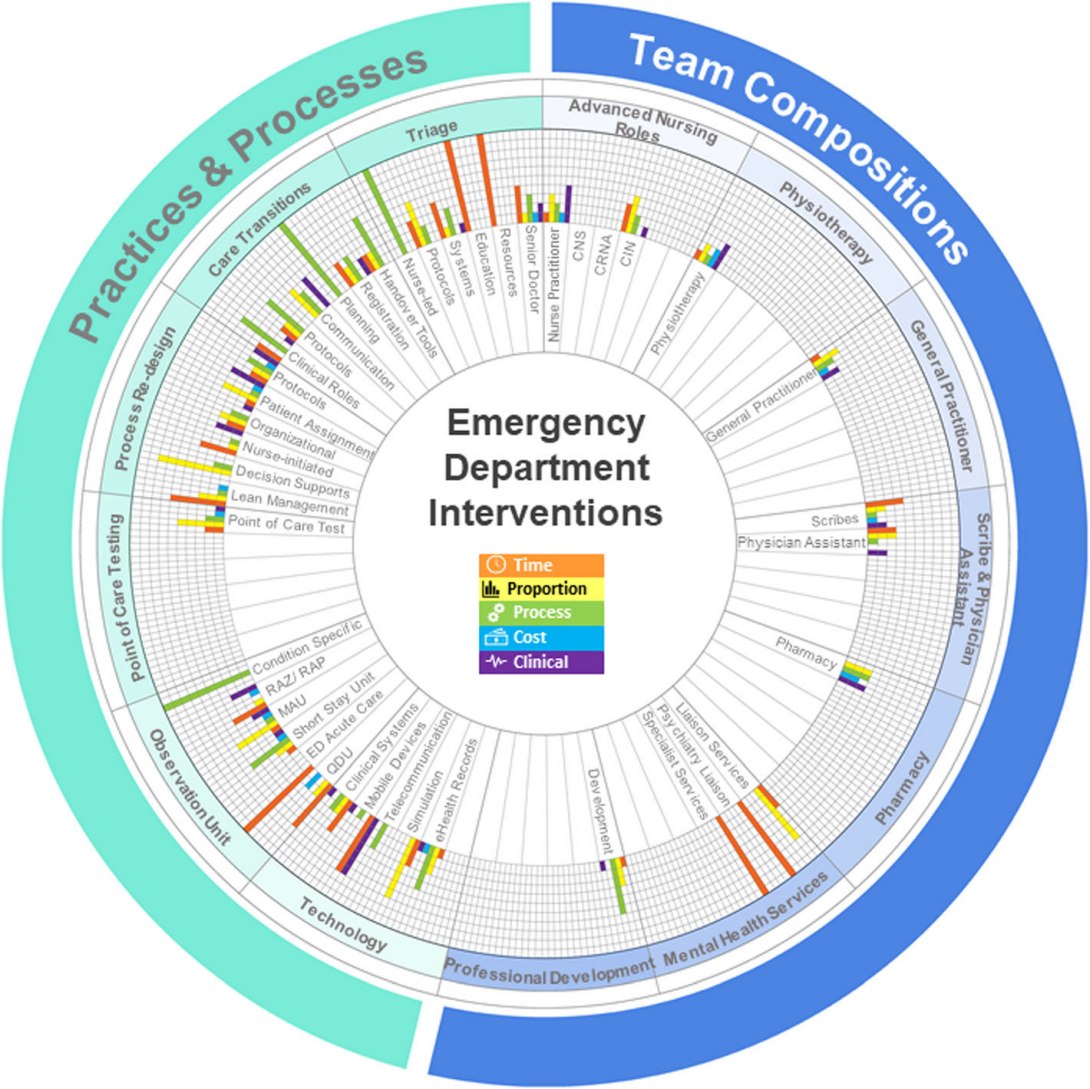
Overview of the outcome measures used for ED interventions. The rich picture summarises the types of interventions identified by this review. The graphs for each intervention present the proportion of outcome measures reported for each domain for each intervention. Each proportion was calculated as the number of identified outcome measures in the domain divided by the total number of outcome measures for the intervention. CNS, clinical nurse specialist; CRNA, certified registered nurse anaesthetist; CIN, clinical initiatives nurse; RAZ/RAP, rapid assessment zone/ rapid assessment pod; MAU, medical assessment unit; ED, Emergency Department; QDU, quick diagnosis unit

The time-based outcome was the most widely used domain for team composition interventions, with 10 of the 13 interventions reporting at least one time-based measure. Proportion-based measures were reported for nine of the 13 interventions with process-based and clinical-based measures reported for 8 of the interventions. Cost-based outcomes were the least utilised, with six interventions reporting at least one outcome in this domain. An additional file provides a full list of intervention performance measures reported for the included interventions (See Additional file 6). Figure 5 displays a summary of the types of interventions within practices and processes and team composition, as well as graphically presenting the proportion of the total number of outcome measures reported for each domain for each intervention.

## Discussion

In this review we aimed to map the research evidence of strategies to measure and improve ED performance. There was strong alignment between how ED performance is measured, the types of ED interventions implemented, and the outcome measures used to assess effectiveness of those interventions.

While EDs worldwide may share a common purpose [87–89], the differences and complexity within each ED system is reflected in the vast number of measures used to understand different aspects of ED performance. Similarly, the different ways these measures have been categorised reflects differences in the interpretation of that common purpose. For EDs and the communities they serve, the selection of performance measures is critical to ensuring a comprehensive, accurate and precise picture of ED performance is developed. It is equally important to develop a shared understanding how ED performance data is collected to ensure that measures used for performance assessment or comparison are valid.

The results of our review show that the delivery of care in ED has evolved over the last 20 years with the implementation of a wide range of interventions to improve ED performance. The interventions identified by this review address very specific aspects of how care is provided in ED, suggesting that a systems perspective has not been applied. Crucially, EDs are complex adaptive systems and any intervention implemented to improve performance is likely influenced by existing models of care, as well as a variety of contextual factors such as funding, availability of skilled workforce, and the physical space available.

Changing patient involvement in the provision of care also plays a role in ED performance. The small number of reviews identified by this review that involved patient perspectives suggests that care delivery in the ED is likely driven by clinicians and protocols, with patients as passive consumers of care. In the crowded and frantic ED context, achieving patient-centred care is likely a challenging task [33]. Our findings suggest that achieving active participation by patients in ED care delivery is possible, but more research is needed on the implications for ED performance and patients’ clinical and psychosocial outcomes.

Intervention outcome measures allow us to determine if the intervention to improve ED performance was successful or if it had unintended outcomes. While the use of all five types of outcome measures synthesised in our review would provide clinicians, hospital administrators and researchers with the most insight into ED performance and intervention effectiveness, implementation of the full suite of measures may not be possible in some contexts. Most studies reported the use of three or fewer types of outcome measures. Measures of time were commonly combined with proportion or process measures. The use of time, proportion and process measures provides insight into the speed of healthcare provision, the quantity of resources used (e.g., diagnostic tests), and the quality of patient management (e.g., clinical documentation). However, intervention implementation decisions are often made based on department budgets or the availability of funding. As such, the inclusion of cost measures is increasingly important to inform clinicians’ and administrators’ decisions about ED performance and intervention effectiveness. Finally, measures of clinical outcomes are also important for examining the assumption that system changes in healthcare provide improved patient safety and clinical outcomes, and this is a neglected area for many interventions.

### Limitations

This scoping review is the first, to our knowledge, to synthesise the many review articles to comprehensively describe the different strategies that have been used to measure and improve ED performance. Limitations of the current study include our pragmatic choice to only include reviews published in English and the potential biases of the included studies. The published reviews examining the effectiveness of interventions in the ED context might have suffered from publication bias, with negative results less likely to be published. As a result of this publication bias, it is unclear what interventions are unsuccessful or if particular context characteristics result in unsuccessful interventions, or negatively impact on patient care.

## Conclusion

Over the last two decades, the way care has been delivered in ED has changed dramatically in response to increased demand and increasing complexity, and it is likely that it will continue to change over the next two decades. In turn, the way we measure ED performance has changed with our capacity to collect and analyse data. We need to think critically about the performance measures we use to define ED performance to ensure we are capturing a complete and dynamic picture that accurately reflects how an ED is performing. As shown by this review, a number of different strategies have been used to improve ED performance. As both internal and external pressures on ED continue to grow, future intervention initiatives will be needed to ensure the tragic consequences of crowding in ED are avoided. Crucially, a comprehensive range of meaningful outcome measures for interventions needs to be used to accurately establish the effectiveness of ED interventions and inform system changes and decision-making.

## Supplementary information

**Additional file 1: Appendix A.** The full search strategy for all databases.

**Additional file 2: Table 1.** Characteristics of the included reviews on ED performance measures.

**Additional file 3: Table 2.** Characteristics of the included reviews on interventions to improve ED performance.

**Additional file 4: Table 3.** The evidence contribution to the review questions of the included reviews.

**Additional file 5: Table 4.** The ROBIS Risk of Bias results for each domain and the overall risk of bias for the included reviews

**Additional file 6: Table 14.** The performance measures reported by the included reviews for each intervention.

## Data Availability

All data generated or analysed during this study are included in this published article and its supplementary information files.
