# Supplementary material for: Strategies to measure and improve emergency department performance: a scoping review
Source: Scand J Trauma Resusc Emerg Med. 2020 Jun 15;28:55. doi: 10.1186/s13049-020-00749-2 (PMC7296671; doi:10.1186/s13049-020-00749-2)
Supplement: Supplementary file 1 — Additional file 1: Appendix A. The full search strategy for all databases. [file 13049_2020_749_MOESM1_ESM.docx]

**Appendix A. Full search strategy**

**Database: Wiley Cochrane Library Database of Systematic Reviews via Macquarie University Library**

'emergency department’ in Title Abstract Keyword

**Database: Elsevier Scopus via Macquarie University Library**

TITLE-ABS-KEY("emergency department*" OR “hospital emergency unit*” OR “hospital emergency service*”) AND TITLE-ABS-KEY("quality" OR "performance" OR "improvement" OR "quality improvement") AND TITLE("review")

**Database: OVID EMBASE via Macquarie University Library**

#1: emergency department.mp

#2: emergency ward.de

#3: #1 OR #2

#4: health care quality.de

#5: quality.ti,ab

#6: improvement.ti,ab

#7: performance.ti,ab

#8: #4 OR #5 OR #6 OR #7

#9: #3 AND #8

#10: review.ti

#11: systematic review.ti

#12: meta-analysis.ti

#13: meta-synthesis.ti

#14: scoping review.ti

#15: integrative review.ti

#16: overview.ti

#17: umbrella review.ti

#18: #10 OR #11 OR #12 OR #13 OR #14 OR #15 OR #16 OR #17

#19: #9 AND #18

**Database: EBSCOhost CINAHL Complete via Macquarie University Library**

(((((MH "emergency Service, Hospital+")) OR "emergency department")) AND ((((((MH "quality of health care+")) OR (MH "quality improvement+")) OR quality) OR improvement) OR performance)) AND ((((((((TI review) OR TI "systematic review") OR TI meta-analysis) OR TI meta-synthesis) OR TI "scoping review") OR TI "integrative review") OR TI overview) OR TI "umbrella review")

**Database: PubMed via Macquarie University Library**

((((emergency Service, Hospital[mh]) OR emergency department[tw])) AND (((((quality of health care[mh]) OR quality improvement[mh]) OR quality[tw]) OR improvement[tw]) OR performance[tw])) AND ((((((((review[ti]) OR systematic review[ti]) OR meta-analysis[ti]) OR meta-synthesis[ti]) OR scoping review[ti]) OR integrative review[ti]) OR overview[ti]) OR umbrella review[ti])
