# Supplementary material for: Strategies to measure and improve emergency department performance: a scoping review
Source: Scand J Trauma Resusc Emerg Med. 2020 Jun 15;28:55. doi: 10.1186/s13049-020-00749-2 (PMC7296671; doi:10.1186/s13049-020-00749-2)
Supplement: Supplementary file 2 — Additional file 2: Table 1. Characteristics of the included reviews on ED performance measures. [file 13049_2020_749_MOESM2_ESM.docx]

**Table 1. Characteristics of the included reviews on ED performance measures**

| **Author, Year, Country** | **Type of review** | **Aim** | **Period of Study** | **No. primary studies/ No. participants** | **ED Performance Measures Summary** |
| --- | --- | --- | --- | --- | --- |
| Madsen, 2015, Denmark | Systematic review | To perform a comprehensive systematic review of emergency department performance indicators in relation to evidence | Not specified | 127 studies/ Sample size not reported | The top 10 indicators by ‘counts’ of evidence were patient satisfaction, standard of care treatment, correct diagnosis, ED occupancy/crowding, time to treatment, ED LOS/wait, ED returns, LWBS and time to diagnosis. |
| Sørup, 2013, Denmark | Umbrella review | To describe, map, and critically evaluate which performance measures that the published literature regard as being most relevant in assessing overall ED performance. | 2002 - 2012 | 14 studies/ Sample size not reported | 55 different performance measures are highlighted. Time intervals and patient related measures were dominant. LOS, time between patient arrival to initial clinical assessment, and time between patient arrivals to admission were highlighted by the majority of articles. LWBS, unplanned re-attendance within a maximum of 72 hours, mortality/morbidity, and number of unintended incidents were the most highlighted performance measures that related directly to the patient. |
| Stang, 2015, Canada | Systematic review | To identify existing measures of ED crowding that have been linked to quality of care as defined by the Institute of Medicine quality domains (safe, effective, patient-centred, efficient, timely, and equitable). | 2004 - 2012 | 32 studies/ 14,798,833 ED patients | The measures most frequently linked to care quality included total ED volume, the number of patients in the waiting room, ED occupancy (percentage of overall beds filled), ED LOS, total patient care hours (sum of the LOS in hours of all patients in the ED), number of admitted patients in the ED awaiting an inpatient bed, and the LOS (in ED) for admitted patients. |
| ED, Emergency Department; ED LOS, Emergency Department length of stay; LWBS, left without being seen; LOS, length of stay; | | | | | |
