# Supplementary material for: Strategies to measure and improve emergency department performance: a scoping review
Source: Scand J Trauma Resusc Emerg Med. 2020 Jun 15;28:55. doi: 10.1186/s13049-020-00749-2 (PMC7296671; doi:10.1186/s13049-020-00749-2)
Supplement: Supplementary file 3 — Additional file 3: Table 2. Characteristics of the included reviews on interventions to improve ED performance. [file 13049_2020_749_MOESM3_ESM.docx]

**Table 2. Characteristics of the included reviews on interventions to improve ED performance**

| **Author, Year, Country** | **Type of review** | **Aim** | **Period of Study** | **No. primary studies/ No. participants** | **Intervention** | **Outcome Assessed** |
| --- | --- | --- | --- | --- | --- | --- |
| **Triage Interventions** | | | | | | |
| Abdulwahid, 2016, England | Systematic review | To determine if single doctor versus single nurse at triage improves ED performance | 1994 – 2014 | 25 studies/ 681,749 medium acuity patients | Senior doctor triage | LOS, waiting time, LWBS, and left without treatment complete |
| Chhabra, 2019, Canada | Systematic review | To identify ED interventions that lead to improvement in door-to- ECG times for adults presenting with symptoms suggestive of acute coronary syndrome | 1993 – 2017 | 11 studies/ 15,622 patients. Sample size not reported for 1 study | Multiple interventions: a dedicated ECG machine and technician in triage (5/11); improved triage education (4/11); improved triage disposition (2/11); and data feedback mechanisms (2/11) | Door-to-ECG time |
| Elder, 2015, Australia | Systematic review | To explore the literature regarding three key strategies designed to promote patient throughput in the ED | 1996 – 2013 | 21 studies/ 105,413 ED staff, patients. Sample size not reported for 1 study | Models of care: expanding nursing roles (e.g., ED NP, nurse- initiated x-ray, CIN), physician-assisted triage, medical assessment units | NP: wait times, unexpected representations, time to be seen by NP, ED LOS; Nurse initiated care: DNW rate, ED LOS, wait times, time to diagnosis, time to treatment, time to analgesia; Physician assisted triage: ED LOS, patient satisfaction, time to laboratory and diagnostic imaging, time to consultation, representation within 48 hours, DNW rates, time to review and disposition. Medical Assessment Unit: ED LOS, wait times for inpatient beds, number of patients waiting in ED for inpatient beds, admissions to medical wards, ED occupancy, overall inpatient days, cost saving, representation rate, readmission rate |
| Harding, 2011, Australia | Systematic review | To answer the following question: Do triage systems across a broad spectrum of health services affect patient flow? | 1992 – 2008 | 25 studies/ 23,710 Patients attending ED; 10,058 other patients | Triage systems: Triage or prioritisation was defined as any system that either ranked patients in order of priority, or sorted patients into the most appropriate service. Doctor at triage | Waiting time, LOS, triage time, LWBS, transit time, DNW, DNA rates, adverse events, number on waiting list |
| McCaughey, 2015, USA | Literature review | To review and synthesize the current academic literature regarding capacity management that focuses on the ED, identify strengths and weaknesses of the approaches in the literature, and provide practical recommendations for hospital administrators | 2001 – 2012 | 23 studies/ Sample size not reported | Bed management scheduling, triage protocols, cross-training nurses, outsourcing environmental services, fast-track, treatment protocols | ED indicators (volume, LOS, Admission rate), Lost business (LWBS, transfer centre declines), Staff efficiency (Environmental services turn-around time, transport), time stamps (door-to-provider time, turn-around time, order-to-results time), patient satisfaction |
| Ming, 2016, Hong Kong | Systematic review and Meta-Analysis | To determine whether ED team triage improves patient flow in comparison with single nurse triage | 2004 – 2013 | 4 studies/ 14,772 patients | Team triage: A triage performed by a team composed of at least two medical personnel, either a nurse or physician | ED LOS, Waiting times, mortality, patient disappearance, patient reattendance |
| Morley, 2018, Australia | Systematic review | To critically analyse and summarise the findings of peer-reviewed research studies investigating the causes and consequences of, and solutions to, emergency department crowding | 2001 - 2017 | 102 studies/ 34,010,814 patients, ED staff | Physicians in triage, Fast-track/ flexible care area; nurse flow coordinator; bedside registration; nurse-initiated protocols; increased bed numbers; timed patient disposition/ discharge targets, point of care testing | Physicians in triage, Fast-track/ flexible care area (wait time, ED LOS, LWBS), point-of-care testing (ED LOS), ED nurse flow coordinator (proportion of patients meeting NEAT, ED occupancy, ambulance transfer of care time, LWBS, ED LOS, ambulance diversion time (hours), bedside registration (Time from triage to room, time from room to disposition, ED LOS, LWBS), nurse initiated protocols (time to diagnostic test, time to treatment, ED LOS), increased bed numbers (LWBS, daily boarding hours), timed patient disposition/ discharge targets (ED LOS, staff perception, proportion of patients leaving ED within 4 hours, mean transit times within the ED, mortality, LWBS rates, 48 hour representation, Inpatient LOS, proportion of admissions meeting NEAT, access block, ED occupancy rate, reattendances/ representation, time to first ED clinician review, readmission, NEAT compliance ), point of care testing (EDLOS) |
| Oredsson, 2011, Sweden | Systematic review | To identify and evaluate the scientific evidence of various interventions to improve patient flow in EDs | 1966 – 2009 | 33 studies/ 489,272 ED patients. Sample size not reported for all studies | Streaming, fast track, team triage, point-of-care testing, nurse-requested x-ray | Fast track: waiting time, ED LOS, proportion of LWBS, patient satisfaction. Team triage: waiting time, ED LOS, proportion of LWBS. Point-of-care testing: Response time, LOS. Nurse requested x-ray: Wait time, LOS |
| Rehman, 2016, England | Systematic review | To determine the factors that affect patient satisfaction with nurse-led-triage in EDs using a systematic review. | 1992 – 2012 | 18 studies/ 106,898 ED patients seen by ENP, ANP, ACP, NP, doctors | Nurse-led-triage services | Patient satisfaction |
| Robinson, 2013, USA | Integrative review | To identify the effectiveness of using triage protocols to decrease ED length of stay | 1992 – 2010 | 8 studies/ 20,848 adult ED patients | Triage protocols | Total LOS in relation to acuity level, LOS after provider assessment, rate of unnecessary radiographs requested, rate of radiographs requested after assessment, rate of positive abnormalities on additional radiographs, satisfaction |
| Rowe, 2011, Canada | Systematic review | To examine the effectiveness of triage liaison physicians on mitigating the effects of ED overcrowding | 1999 – 2009 | 28 studies/ 406,184 adult and child patients. Sample size not reported for all studies | Triage liaison physicians: physicians work with triage (a system of sorting patients based on acuity and risk) staff to expedite the care of patients | ED LOS, physician initial assessment time, LWBS, leaving against medical advice |
| Wylie, 2015, Australia | Systematic review | To identify current ED models of care and their impact on care quality, care effectiveness, and cost | Period of study not reporter | 66 studies/ Sample size not reported | ED models of care: NP role, physician in triage, physician assistants and ED scribes, Care coordination teams, Fast-Track, rapid assessment units | NP (wait time, LOS, patient satisfaction, errors, adverse events, representation rates, staff turnover), physician in triage (LOS, ambulance diversions, ), physician assistants and ED scribes (LOS), Care coordination teams (unnecessary social admission, re-presentation, staff satisfaction, retention rate), Fast-Track (wait time, LOS, costs, off stretcher times, ambulance diversion rates) |
| **Care Transitions (Handover processes) Interventions** | | | | | | |
| Alimenti, 2019, USA | Systematic review | To analyse existing literature pertaining to handoffs between the ED and inpatient setting, and its effect on perceived patient safety | 2011 - 2015 | 4 studies/ 245 preintervention and 1228 postintervention participants including ED and internal medicine physicians, nurses and registrars | Hand off tool | Perceptions of patient safety, factors related to patient safety in the handoff process |
| Boudreaux, 2006, USA | Critical review | To determine if Performance Improvement methods, in general, can be used to improve ED patient satisfaction, and which specific process changes or interventions show the most promise, either by way of head-to-head comparisons, larger effect sizes, or replication across different settings. | 1996 - 2004 | 19 studies/ 9,017 ED patients | Performance Improvement interventions: change to clinical practice guidelines, bedside registration, pathology protocols, patient assignment processes, customer service training, discharge planning, referral processes, ED Observation Units | Patient reported problems or patient complaints, patient satisfaction, patient waiting time satisfaction, Picker Institute Survey, patient rated nursing/doctor physician bedside manner/ technical skill |
| Curran, 2019, Canada | Systematic review | To examine how and why discharge communication works in a paediatric ED context and develop recommendations for practice, policy, and research | 1979 - 2018 | 75 studies/ 13,187 parents, children, adolescents, patients, ED physicians. 2563 patient charts/ medical records. Sample size not reported for all studies | Discharge communication interventions | Parent recall of discharge information, knowledge and comprehension, provider behaviour, adherence to guidelines, unnecessary ED return visits, discharge advice, knowledge acquisition, asthma management |
| Dawson, 2013, Australia | Integrative review | To establish: (i) what aspects of the clinical handover between paramedics and ED staff impact on the effective transfer of a patient in a state of physiological deterioration; and (ii) how these aspects might be improved in the future | 2001 – 2012 | 17 studies/ ~2000 aged care staff, ED medical officers, ED nurses, patients, paramedics | Paramedic handover (processes, protocols, and tools) to ED | Quality of handover, perception of utility, types and characteristics of handover, information recall |
| Flynn, 2016, England | Systematic review | To summarise the current evidence describing the impact of enhanced paramedic processing of emergency conditions with time-dependent treatment outcomes (i.e. trauma, stroke and MI) | 1994 - 2016 | 36 studies/ Paramedics EMTs, ambulance technicians, trauma patients. Sample size not reported for each study | Structured handover tools/ protocols, protocols and enhanced skills training to improve handover, and protocols or enhanced skills leading to a change in in-hospital transfer location | Physician recall, documentation quality, guideline adherence, door-to-CT scan time |
| Morley, 2018, Australia | Systematic review | To critically analyse and summarise the findings of peer-reviewed research studies investigating the causes and consequences of, and solutions to, emergency department crowding | 2001 - 2017 | 102 studies/ 34,010,814 patients, ED staff | Physicians in triage, Fast-track/ flexible care area; nurse flow coordinator; bedside registration; nurse-initiated protocols; increased bed numbers; timed patient disposition/ discharge targets, point of care testing | Physicians in triage, Fast-track/ flexible care area (wait time, ED LOS, LWBS), point-of-care testing (ED LOS), ED nurse flow coordinator (proportion of patients meeting NEAT, ED occupancy, ambulance transfer of care time, LWBS, ED LOS, ambulance diversion time (hours), bedside registration (Time from triage to room, time from room to disposition, ED LOS, LWBS), nurse initiated protocols (time to diagnostic test, time to treatment, ED LOS), increased bed numbers (LWBS, daily boarding hours), timed patient disposition/ discharge targets (ED LOS, staff perception, proportion of patients leaving ED within 4 hours, mean transit times within the ED, mortality, LWBS rates, 48 hour representation, Inpatient LOS, proportion of admissions meeting NEAT, access block, ED occupancy rate, reattendances/ representation, time to first ED clinician review, readmission, NEAT compliance ), point of care testing (EDLOS) |
| Reay, 2019, Canada | Systematic review | To examine (1) factors that influence transitions in care from EMS providers to ED nurses and (2) the effectiveness of interventional strategies to improve these transitions | 2003 - 2017 | 20 studies/ 1,056 EMS providers (paramedics, emergency medical technicians, registered nurses, licensed practical nurses, nurse practitioners), ED nurses (registered nurses, licensed practical nurses, nurse practitioners), physicians, patients. Sample size not reported for all studies | Three interventions were identified in 6 methodologically weak studies: (1) transition guideline (DeMIST, Identification, Mechanism/Medical complaint, Injuries/ Information related to the complaint, Signs, Treatment and Trends – Allergies, Medication, Background history, Other information [IMIST-AMBO]) with training, (2) mobile web-based technology (EMS smartphone and geographic information system location data), and (3) a new clinical role (ED ambulance off-load nurse dedicated to triaging and assessing EMS patients) | Guideline use and adherence, communication between EMS providers and ED nurses, effectiveness of the transition, wait times, time to be seen, LWBS, LOS |
| Shankar, 2014, USA | Systematic review | To synthesize the current knowledge about the elderly patient’s preferences and views of their emergency care | 1984 - 2012 | 28 studies/ 783,917-783,957 elderly adult patients (>=65 years) receiving emergency care | Nurse discharge coordinators | Patient satisfaction, wait times, unscheduled admissions within 14 days of discharge |
| **Process Re-design Interventions** | | | | | | |
| Bennett, 2017, England | Substantive review | To identify the body of computerised clinical decision support systems research undertaken in EDs, the re- search methods used, their quality, and the impact of computerised clinical decision support systems on clinical care in EDs | 1995 - 2013 | 23 studies/ 23,871 patients with a variety of clinical conditions. Sample not specified for 2 studies | Computerised clinical decision support systems | Time to measurement (various), admission rate, ED LOS, timeliness of tests/imaging, quality of care, cost of care, number of scans ordered |
| Boudreaux, 2004, USA | Systematic review | To answer two questions: 1) what are the strongest predictors of ED patient satisfaction? and 2) what changes can be made to improve ED patient satisfaction? | 1993 - 2002 | 50 studies/ 31,920 patients. Sample not specified for 37 studies | Patient satisfaction interventions: feedback of QI data and setting of benchmarks, videotape providing information about ED, MD/ RN patient care teams, information hand out, customer service training, 10 week medical Spanish course, chest pain observation unit, asthma observation unit, MD business cards, TVs in rooms, process redesign | Patient satisfaction with ED care |
| Boudreaux, 2006, USA | Critical review | To determine if Performance Improvement methods, in general, can be used to improve ED patient satisfaction, and which specific process changes or interventions show the most promise, either by way of head-to-head comparisons, larger effect sizes, or replication across different settings | 1996 - 2004 | 19 studies/ 9,017 ED patients | Performance Improvement interventions: change to clinical practice guidelines, bedside registration, pathology protocols, patient assignment processes, customer service training, discharge planning, referral processes, ED Observation Units | Patient reported problems or patient complaints, patient satisfaction, patient waiting time satisfaction, Picker Institute Survey, patient rated nursing/doctor physician bedside manner/ technical skill |
| Bucci, 2016, Italy | Systematic review | To provide a synthesis of the current literature focused on how Lean Thinking Principles and tools can be applied in an ED to address overcrowding and hospital admissions | 2006 - 2014 | 9 studies/ ED staff including clinicians, nurses, lean consultants | Lean Thinking applied to EDs using lean tools and processes. Can include process redesign, changing roles, and implementing technology | Patient volume, LOS, number of admitted patients, direct expense, patient satisfaction, number of patients discharged, median exam room time, LWBS, number of patients seen and discharged within 4 hours, waiting time |
| Cabilan, 2017, Australia | Systematic review | To systematically review the literature evaluating the effects of nurse-initiated medications in the emergency department; and to quantify the impact of the practice on quality of care indicators (i.e. safety, timeliness, effectiveness, equitability, patient-centred care, and efficiency) | 2010 - 2016 | 5 studies/ 1272 patients: paediatric (352) and adult (920) patients who sought care in the ED | Nurse-initiated medication | Safety (nausea and vomiting, medication errors, perception of tremors and palpitation, and ED representations, deviation of vital signs), timeliness (time to bronchodilators, time to analgesia), effectiveness (clinical improvement, clinically relevant pain relief), equitability (access to analgesia), patient-centeredness (patient satisfaction), and efficiency (ED LOS, doctor wait time) |
| Considine, 2019, Australia | Systematic review | To determine the effectiveness of nurse-initiated X-ray for ED patients with distal limb injuries | 1971 - 2018 | 16 studies/ 8881 patients with distal limb injuries, nurses and medical officers | Nurse initiated x-ray | X-ray request practices, accuracy of X-ray requests, time to X-ray, waiting time (defined as the time from triage to medical assessment), ED LOS (defined as the time from triage to ED discharge), patient satisfaction |
| Deblois, 2018, Canada | Systematic review | To summarize the evidence associated with the interventions aimed at reducing the overuse of imaging in the diagnostic workup of Pulmonary Embolism in the ED and hospital wards | 2001 – 2017 | 17 (15 ED) studies/ 32,938 patients, ED physicians | Clinical Decision Support, Educational intervention, Performance and Feedback Reports, Policy | Imaging use, diagnostic yield, adherence to guidelines |
| De Freitas, 2018, England | Umbrella review | To summarise the evidence from systematic reviews on the interventions that improve patient flow in EDs | Reviews: 2006 - 2016 | 13 studies/ 2,225,339 ED staff, patients | Senior doctor triage, diagnostic services, assessment/short stay units, nurse directed interventions, administrative/organisational- e.g., scribes, and computerised provider order entry | Senior doctor triage (ED LOS, waiting time),diagnostic services, assessment/short stay units (ED LOS, Waiting time, physician initial assessment time), nurse directed interventions (ED LOS, patient off stretcher times), administrative/organisational- e.g., scribes, (ED LOS, waiting times, door to room, room to doctor, time to disposition, patients per hour), and computerised provider order entry (ED LOS) |
| Desai, 2018, Canada | Systematic review | To identify, describe the implementation strategies, and examine the effectiveness of evidence-based interventions (such as Clinical Decision Rules or guidelines) aimed at reducing C-spine image ordering in alert adult patients presenting to the ED with neck injury | 1995 - 2009 | 7 studies/ 54,115 stable adult patients (≥17 years) presenting to an ED or other acute care centre with neck trauma | Evidence-based tools such as clinical practice guidelines and Clinical Decision Rules | Changes in image ordering, intervention fidelity |
| Elder, 2015, Australia | Systematic review | To explore the literature regarding three key strategies designed to promote patient throughput in the ED | 1996 - 2013 | 21 studies/ 105,413 ED staff, patients. Sample size not reported for 1 study | Models of care: expanding nursing roles (e.g., ED NP, nurse- initiated x-ray, CIN), physician-assisted triage, medical assessment units | NP: wait times, unexpected representations, time to be seen by NP, ED LOS; Nurse initiated care: DNW rate, ED LOS, wait times, time to diagnosis, time to treatment, time to analgesia; Physician assisted triage: ED LOS, patient satisfaction, time to laboratory and diagnostic imaging, time to consultation, representation within 48 hours, DNW rates, time to review and disposition. Medical Assessment Unit: ED LOS, wait times for inpatient beds, number of patients waiting in ED for inpatient beds, admissions to medical wards, ED occupancy, overall inpatient days, cost saving, representation rate, readmission rate. |
| Holden, 2011, USA | Critical review | To critically review and analyse the empirical literature on the implementation of Lean in the ED | 2006 - 2010 | 18 studies/ Health care employees in addition to patients. The staff involved ranged from clinicians to clerks, assistants, engineers, and representatives of the patient community. Sample size not reported for all studies | Lean thinking concepts, methods, and tools | LOS, waiting times, and proportion of patients leaving the ED without being seen, patient satisfaction |
| Hughes, 2019, USA | Systematic review | To evaluate the effect of ED interventions on clinical, utilization, and care experience outcomes for older adults | Period of study was not reported | 17 studies/ 16,141 Older adults 65 years of age and older | Interventions for older adults using the ED: discharge planning, case management, medication safety or management, and geriatric EDs including those that cited the 2014 Geriatric ED Guidelines | functional status, QOL, patient experience, hospitalization at or after the initial ED index visit, or ED return visit |
| Hoot, 2008, USA | Systematic review | To describe the scientific literature on ED crowding from the perspective of causes, effects, and solutions | 1991 - 2006 | 93 studies/ >72,000 ED patients. Sample size not reported for all studies | Increased resources (additional personnel, observation units), demand management (nonurgent referrals), operations research (crowding measures and offline change management techniques), administrative interventions | Increased resources (additional personnel, observation units) LOS, ambulance diversion, LWBS, number of patients boarding, demand management (nonurgent referrals) ambulance diversion, need for diversion, ambulance arrivals, ED LOS |
| Isfahani, 2019, Iran | Systematic review | To review effectiveness of Lean management strategies in EDs | 2000 - 2016 | 26 studies/ ED physicians, nurses, senior leaders, hospital management team, and other staff members. Sample size not reported for all studies | Lean management interventions | ED LOS, time to physician assessment, login to disposition time, login to triage time, waiting time, lead time, triage waiting time, waiting time for consultation, admission waiting time, registration to physician time, time spent in the examining area by patients, cost per patient, proportion of cases with 12 lead ECG completed within 10 minutes of triage, physician assessment initiated within 60 minutes, time to ECG, time to physician assessment, time to ASA administration, door to doctor time, LWBS, number of searches of supplies by nurses, number of patients discharges in <1hr, patient satisfaction |
| Jones, 2010, New Zealand | Systematic review | To determine what effects the 4-hour time target for ED LOS had on clinically relevant outcomes in the UK | 2005 - 2010 | 8 studies/ 99 nurses, patients, families, ED consultants. Sample size not reported for all studies | The 4-hour rule: mandates that 98% of ED patients are discharged or admitted within 4 h of arriving in ED | Time to clinician, tests per patient, admissions, admissions <24hr, DNW, mortality, return <7 days, treated <1hr, presentations, surgery wait times, attitudes/beliefs about 4hr target |
| Juillard, 2009, USA | Systematic review | To provide a more comprehensive description of the evidence base that supports the global efforts to increase the use of trauma QI programs | 1986 - 2008 | 36 studies/ Trauma patients. Sample size not reported for all studies | QI programs. In the ED: trauma radiology: ultrasound program, communication and consultation, redundant reading of emergency CT scans | Change in mortality, infection rate, patient satisfaction, average length of hospital stay, cost saving, error rate, adverse events, patient call-backs, time to diagnostic test, turn-around time, patient volume, complaints, left against medical advice, change in processes of care |
| Kirkland, 2019, Canada | Systematic review | To examine the effectiveness and safety of pre-hospital and ED-based diversion strategies on ED utilisation, non-ED healthcare utilisation and patient outcomes compared with standard emergency care responses | 2000 - 2017 | 15 studies/ 17,037 patients with nonserious concerns, low-acuity concerns, minor injury or illness, falls, any, abdominal pain, musculoskeletal concerns seeking care in ED | Interventions designed to either bypass the ED or direct patients to other alternative care after ED presentation | ED attendance, subsequent ED attendance, use of other healthcare services, hospitalisation, patient outcomes, cost-effectiveness |
| Kumar, 2013, USA | Systematic review | To examine the evidence of the effectiveness of the case management model in the frequent ED user patient population | 1996 - 2011 | 12 studies/ 960 patients >18years identified as frequent ED users without specific limitations on medical condition, reason for ED utilization, or complaint | Case management interventions | ED utilization, inpatient admission rates, cost, and psychosocial outcomes |
| McCaughey, 2015, USA | Systematic review | To review and synthesize the current academic literature regarding capacity management that focuses on the ED, identify strengths and weaknesses of the approaches in the literature, and provide practical recommendations for hospital administrators | 2001 - 2012 | 23 studies/ Sample size not reported | Bed management scheduling, triage protocols, cross-training nurses, outsourcing environmental services, fast-track, treatment protocols | ED indicators (volume, LOS, Admission rate), Lost business (LWBS, transfer centre declines), Staff efficiency (Environmental services turn-around time, transport), time stamps (door-to-provider time, turn-around time, order-to-results time), patient satisfaction |
| Mieiro, 2019, Brazil | Integrative review | To assess the strategies used by the Nursing team to minimize medication errors in emergency units | 2005 - 2016 | 3 studies/ 1744 sample size not reported for all studies | Educational strategies (conducting campaigns, elaborating explanatory manuals, creating a multidisciplinary committee involved in the prevention and reduction of adverse drug events); organizational (meetings, deviance positive, creation of protocols and changes in the work process) and new technologies (implementation of prescription by computerized system, introduction of the unit doses and of the bar code in the administration of medicines) | Medication errors |
| Morley, 2018, Australia | Systematic review | To critically analyse and summarise the findings of peer-reviewed research studies investigating the causes and consequences of, and solutions to, emergency department crowding | 2001 - 2017 | 102 studies/ 34,010,814 patients, ED staff | Physicians in triage, Fast-track/ flexible care area; nurse flow coordinator; bedside registration; nurse-initiated protocols; increased bed numbers; timed patient disposition/ discharge targets, point of care testing | Physicians in triage, Fast-track/ flexible care area (wait time, ED LOS, LWBS), point-of-care testing (ED LOS), ED nurse flow coordinator (proportion of patients meeting NEAT, ED occupancy, ambulance transfer of care time, LWBS, ED LOS, ambulance diversion time (hours), bedside registration (Time from triage to room, time from room to disposition, ED LOS, LWBS), nurse initiated protocols (time to diagnostic test, time to treatment, ED LOS), increased bed numbers (LWBS, daily boarding hours), timed patient disposition/ discharge targets (ED LOS, staff perception, proportion of patients leaving ED within 4 hours, mean transit times within the ED, mortality, LWBS rates, 48 hour representation, Inpatient LOS, proportion of admissions meeting NEAT, access block, ED occupancy rate, reattendances/ representation, time to first ED clinician review, readmission, NEAT compliance ), point of care testing (EDLOS) |
| Oredsson, 2011, Sweden | Systematic review | To identify and evaluate the scientific evidence of various interventions to improve patient flow in EDs | 1966 - 2009 | 33 studies/ 489,272 ED patients. Sample size not reported for all studies | Streaming, fast track, team triage, point-of-care testing, nurse-requested x-ray | Fast track: waiting time, ED LOS, proportion of LWBS, patient satisfaction. Team triage: waiting time, ED LOS, proportion of LWBS. Point-of-care testing: Response time, LOS. Nurse requested x-ray: Wait time, LOS |
| Rowe, 2011, Canada | Systematic review | To examine the effectiveness of triage nurse ordering on mitigating the effect of ED overcrowding | 1971 - 2011 | 14 studies/ 24,096 adult (17 years or older) or mixed (child and adult) patients | Triage nurse ordering of x-rays, diagnostic tests | ED LOS (time in minutes from patient ED arrival to departure), time from patient ED arrival to Physician initiated assessment, proportion of radiographs ordered |
| Williams, 2019, Australia | Integrative literature review | To explore systems level change in the ED for improved paediatric pain management | 2001 - 2016 | 20 studies/ 6,641 patients. sample sizes ranged from 52 to 1,200, with three studies including less than 100 participants | Evidence-based protocols or policy, nurse-initiated analgesia, clinical education, family/ patient involvement | Nurse-initiated analgesia (time to analgesia, provision of analgesia, parental satisfaction), clinical education (provision of analgesia, documentation of pain scores), family/ patient involvement (pain score, pain beliefs, time to analgesia) |
| **Point-of-Care Testing Interventions** | | | | | | |
| Bingisser, 2012, Switzerland | Literature review | To examine the current status of point-of-care testing in the emergency department setting, with regard to its evidence base, limitations, advantages, and barriers, and, in respect of a series of key questions, its impact on length of stay, patient management, outcomes, and resource use. | Period of study not reported. | Number of primary studies including not reported. Number of participants not reported. Patients presenting with chest pain to ED | Point of care testing for cardiac troponin | Reliability of point of care testing for cardiac troponin; impact of point of care testing for cardiac troponin on patient outcome/safety, LOS, cost, and management of patients |
| De Freitas, 2018, England | Umbrella review | To summarise the evidence from systematic reviews on the interventions that improve patient flow in EDs | Reviews: 2006 - 2016 | 13 studies/ 2,225,339 ED staff, patients | Senior doctor triage, diagnostic services, assessment/short stay units, nurse directed interventions, administrative/organisational- e.g., scribes, and computerised provider order entry | Senior doctor triage (ED LOS, waiting time),diagnostic services, assessment/short stay units (ED LOS, Waiting time, physician initial assessment time), nurse directed interventions (ED LOS, patient off stretcher times), administrative/organisational- e.g., scribes, (ED LOS, waiting times, door to room, room to doctor, time to disposition, patients per hour), and computerised provider order entry (ED LOS) |
| Doan, 2014, Canada | Systematic review | To determine if the use of a rapid viral detection test for children with an acute respiratory infection in EDs changes patient management and resource use in the ED, compared to not using a rapid viral detection test | 2003 - 2009 | 4 studies/ 1595 previously health children <18 years who attended an ED or urgent care clinic due to fever or respirator symptoms | Rapid viral diagnostic testing | Antimicrobial prescription rate in the ED, ED LOS, rate of ancillary tests, rate of physician visit within 2 weeks, hospital admission rate, Acceptability of nasal specimen collection sampling for rapid viral testing |
| Morley, 2018, Australia | Systematic review | To critically analyse and summarise the findings of peer-reviewed research studies investigating the causes and consequences of, and solutions to, emergency department crowding | 2001 - 2017 | 102 studies/ 34,010,814 patients, ED staff | Physicians in triage, Fast-track/ flexible care area; nurse flow coordinator; bedside registration; nurse-initiated protocols; increased bed numbers; timed patient disposition/ discharge targets, point of care testing | Physicians in triage, Fast-track/ flexible care area (wait time, ED LOS, LWBS), point-of-care testing (ED LOS), ED nurse flow coordinator (proportion of patients meeting NEAT, ED occupancy, ambulance transfer of care time, LWBS, ED LOS, ambulance diversion time (hours), bedside registration (Time from triage to room, time from room to disposition, ED LOS, LWBS), nurse initiated protocols (time to diagnostic test, time to treatment, ED LOS), increased bed numbers (LWBS, daily boarding hours), timed patient disposition/ discharge targets (ED LOS, staff perception, proportion of patients leaving ED within 4 hours, mean transit times within the ED, mortality, LWBS rates, 48 hour representation, Inpatient LOS, proportion of admissions meeting NEAT, access block, ED occupancy rate, reattendances/ representation, time to first ED clinician review, readmission, NEAT compliance ), point of care testing (EDLOS) |
| Oredsson, 2011, Sweden | Systematic review | To identify and evaluate the scientific evidence of various interventions to improve patient flow in EDs | 1966 - 2009 | 33 studies/ 489,272 ED patients. Sample size not reported for all studies | Streaming, fast track, team triage, point-of-care testing, nurse-requested x-ray | Fast track: waiting time, ED LOS, proportion of LWBS, patient satisfaction. Team triage: waiting time, ED LOS, proportion of LWBS. Point-of-care testing: Response time, LOS. Nurse requested x-ray: Wait time, LOS |
| **Observation Unit Interventions** | | | | | | |
| Boudreaux, 2004, USA | Systematic review | To answer two questions: 1) what are the strongest predictors of ED patient satisfaction? and 2) what changes can be made to improve ED patient satisfaction? | 1993 - 2002 | 50 studies/ 31,920 patients. Sample not specified for 37 studies | Patient satisfaction interventions: feedback of QI data and setting of benchmarks, videotape providing information about ED, MD/ RN patient care teams, information hand out, customer service training, 10 week medical Spanish course, chest pain observation unit, asthma observation unit, MD business cards, TVs in rooms, process redesign | Patient satisfaction with ED care |
| Boudreaux, 2006, USA | Critical review | To determine if Performance Improvement methods, in general, can be used to improve ED patient satisfaction, and which specific process changes or interventions show the most promise, either by way of head-to-head comparisons, larger effect sizes, or replication across different settings | 1996 - 2004 | 19 studies/ 9,017 ED patients | Performance Improvement interventions: change to clinical practice guidelines, bedside registration, pathology protocols, patient assignment processes, customer service training, discharge planning, referral processes, ED Observation Units | Patient reported problems or patient complaints, patient satisfaction, patient waiting time satisfaction, Picker Institute Survey, patient rated nursing/doctor physician bedside manner/ technical skill |
| Bullard, 2012, Canada | Systematic review | To evaluate the effectiveness of a rapid assessment zone to mitigate ED overcrowding | 2001 - 2008 | 4 studies/ 23,189 adult ED or mixed (paediatric & adult) ED patients | Rapid Assessment Zones/Pods | LOS (time in min from patient arrival to departure from the ED), physician initial assessment (time in min from patient arrival to being seen by a doctor), LWBS |
| De Freitas, 2018, England | Umbrella review | To summarise the evidence from systematic reviews on the interventions that improve patient flow in EDs | Reviews: 2006 - 2016 | 13 studies/ 2,225,339 ED staff, patients | Senior doctor triage, diagnostic services, assessment/short stay units, nurse directed interventions, administrative/organisational- e.g., scribes, and computerised provider order entry | Senior doctor triage (ED LOS, waiting time),diagnostic services, assessment/short stay units (ED LOS, Waiting time, physician initial assessment time), nurse directed interventions (ED LOS, patient off stretcher times), administrative/organisational- e.g., scribes, (ED LOS, waiting times, door to room, room to doctor, time to disposition, patients per hour), and computerised provider order entry (ED LOS) |
| Elder, 2015, Australia | Systematic review | To explore the literature regarding three key strategies designed to promote patient throughput in the ED | 1996 - 2013 | 21 studies/ 105,413 ED staff, patients. Sample size not reported for 1 study | Models of care: expanding nursing roles (e.g., ED NP, nurse- initiated x-ray, CIN), physician-assisted triage, medical assessment units | NP: wait times, unexpected representations, time to be seen by NP, ED LOS; Nurse initiated care: DNW rate, ED LOS, wait times, time to diagnosis, time to treatment, time to analgesia; Physician assisted triage: ED LOS, patient satisfaction, time to laboratory and diagnostic imaging, time to consultation, representation within 48 hours, DNW rates, time to review and disposition. Medical Assessment Unit: ED LOS, wait times for inpatient beds, number of patients waiting in ED for inpatient beds, admissions to medical wards, ED occupancy, overall inpatient days, cost saving, representation rate, readmission rate |
| Galipeau, 2015, Canada | Systematic review | To evaluate the effectiveness and safety of ED short-stay units compared with care not involving Short-Stay Units | 1997 - 2013 | 5 studies/ Study size ranged from 105 to 222 adult patients. Sample size not reported for each study | Short-stay units: Short-stay units were defined as general-purpose units, beyond a simple extension of beds, designed to treat and/or observe any ED patients with expected LOS of 72 hours or less | LOS, hospital readmission, total problems, communication, special needs/preferences, Financial information, emotional support, physical comfort, patient education, family involvement, pain management, discharge prep, perceived LOS |
| Goodacre, 2000, England | Systematic review | To examine the evidence for effectiveness and economic efficiency of the Chest Pain Observation Unit and to explore whether data from the United States can be extrapolated to the UK | 1989 - 1999 | 11 studies/ 9,665 patients managed on Chest Pain Observation Unit | Chest Pain Observation Unit | Mortality, missed pathology, adverse events, return visit/ rehospitalisation, cost saving |
| Hoot, 2008, USA | Systematic review | To describe the scientific literature on ED crowding from the perspective of causes, effects, and solutions | 1991 - 2006 | 93 studies/ >72,000 ED patients. Sample size not reported for all studies. | Increased resources (additional personnel, observation units), demand management (nonurgent referrals), operations research (crowding measures and offline change management techniques), administrative interventions | Increased resources (additional personnel, observation units) LOS, ambulance diversion, LWBS, number of patients boarding, demand management (nonurgent referrals) ambulance diversion, need for diversion, ambulance arrivals, ED LOS |
| Konnyu, 2012, Canada | Rapid review | To summarises evidence of the effectiveness and safety of Short Stay Units in the ED | 2003 - 2008 | 4 studies/ 9,961 patients | ED Short Stay Units | Patient satisfaction, LOS, ED efficiency, cost effectiveness |
| McCaughey, 2015, USA | Systematic review | To review and synthesize the current academic literature regarding capacity management that focuses on the ED, identify strengths and weaknesses of the approaches in the literature, and provide practical recommendations for hospital administrators | 2001 - 2012 | 23 studies/ Sample size not reported | Bed management scheduling, triage protocols, cross-training nurses, outsourcing environmental services, fast-track, treatment protocols, | ED indicators (volume, LOS, Admission rate), Lost business (LWBS, transfer centre declines), Staff efficiency (Environmental services turn-around time, transport), time stamps (door-to-provider time, turn-around time, order-to-results time), patient satisfaction |
| Morley, 2018, Australia | Systematic review | To critically analyse and summarise the findings of peer-reviewed research studies investigating the causes and consequences of, and solutions to, emergency department crowding | 2001 - 2017 | 102 studies/ 34,010,814 patients, ED staff | Physicians in triage, Fast-track/ flexible care area; nurse flow coordinator; bedside registration; nurse-initiated protocols; increased bed numbers; timed patient disposition/ discharge targets, point of care testing | Physicians in triage, Fast-track/ flexible care area (wait time, ED LOS, LWBS), point-of-care testing (ED LOS), ED nurse flow coordinator (proportion of patients meeting NEAT, ED occupancy, ambulance transfer of care time, LWBS, ED LOS, ambulance diversion time (hours), bedside registration (Time from triage to room, time from room to disposition, ED LOS, LWBS), nurse initiated protocols (time to diagnostic test, time to treatment, ED LOS), increased bed numbers (LWBS, daily boarding hours), timed patient disposition/ discharge targets (ED LOS, staff perception, proportion of patients leaving ED within 4 hours, mean transit times within the ED, mortality, LWBS rates, 48 hour representation, Inpatient LOS, proportion of admissions meeting NEAT, access block, ED occupancy rate, reattendances/ representation, time to first ED clinician review, readmission, NEAT compliance ), point of care testing (EDLOS) |
| Wylie, 2015, Australia | Systematic review | To identify current ED models of care and their impact on care quality, care effectiveness, and cost | Period of study not reported | 66 studies/ Sample size not reported | ED models of care: NP role, physician in triage, physician assistants and ED scribes, Care coordination teams, Fast-Track, rapid assessment units | NP role (wait time, LOS, patient satisfaction, errors, adverse events, representation rates, staff turnover), physician in triage (LOS, ambulance diversions, ), physician assistants and ED scribes (LOS), Care coordination teams (unnecessary social admission, re-presentation, staff satisfaction, retention rate), Fast-Track (wait time, LOS, costs, off stretcher times, ambulance diversion rates) |
| **Technology Interventions** | | | | | | |
| Bennett, 2017, England | Substantive review | To identify the body of computerised clinical decision support systems research undertaken in EDs, the re- search methods used, their quality, and the impact of computerised clinical decision support systems on clinical care in EDs | 1995 - 2013 | 23 studies/ 23,871 patients with a variety of clinical conditions. Sample not specified for 2 studies | Computerised clinical decision support systems | Time to measurement (various), admission rate, ED LOS, timeliness of tests/imaging, quality of care, cost of care, number of scans ordered |
| Bowden, 2017, Australia | Systematic review | To assess the impact of accessing primary care records on unscheduled care | 2006 - 2015 | 22 studies/ Patients receiving unscheduled care in regions with shared electronic health records. Reported by % of population | Access to electronic health records from primary care during unscheduled care | Clinician utilisation rate |
| Dexheimer, 2015, USA | Scoping review of primary studies | To outline the current state of the research using mobile devices in the ED | 2003 - 2012 | 10 studies/ ED physicians, patients, medical students. Sample size not reported for all studies | Mobile devices such as wireless mobile computers, mobile workstations, personal digital assistants, handheld computers, tablets, or Smartphones in the ED | Access time, user’s perceptions of the speed; efficiencies arising from improved communication, time spent accessing information, improved guideline accuracy, error rate |
| Georgiou, 2013, Australia | Systematic review | To examine evidence of the effect of computerized provider order entry on clinical care and work processes in the ED | 2002 - 2011 | 22 studies/ 61,851 patients and ED staff | Computerised provider entry form: An electronic system used to enter patient data | Time spent on computers, using paper, or patient care; turnaround time; LOS; Time between 2 points (e.g., interval between a patient arriving in the ED and a test being ordered), number of test orders, order sets; compliance or noncompliance with recommendations/guidelines, prescription rate (e.g., of medication considered inappropriate); Time until order renewal; Vaccination rates, medication error rates (e.g., overdose, underdose, excessive dosing, wrong route, wrong dose, wrong drug); specimen processing errors; case fatality rate; time in restraints |
| Hersh, 2015, USA | Systematic review | To review Health Information Exchange and determine needs for future research that reflect our assessment of the benefits and limitations of Health Information Exchange | 2006 - 2015 | 34 studies/ Sample size not reported for all studies. | Health information exchange, defined as the reliable and interoperable electronic sharing of clinical information among physicians, nurses, pharmacists, other health care providers, and patients across the boundaries of health care institutions, health data repositories, laboratories, public health agencies, and other entities that are not within a single organization or among affiliated providers | Lab testing, radiology testing, hospital admissions, LOS, hospital readmissions, referrals and consultations, ED frequent users, ED costs, public heath reporting, quality of care, and other aspects of Health Information Exchange. Patient/ clinician perceptions |
| Kelton, 2018, Canada | Scoping review | To collect and synthesize the literature describing the use of real-time video-based technologies to provide support in the care of patients presenting to emergency departments | 1997 - 2014 | 11 studies/ ED patients. Sample size not reported for all studies | Telemedicine in ED: encompass the use of telecommunications technology (including the transmission of video, still images, radiological studies, physiological data, and pathology results) to provide health care services to a patient | Return <3 days, need further treatment, patient satisfaction |
| Mohiuddin, 2017, England | Systematic review | To investigate the different computer simulation methods and their contribution for analysis of patient flow within EDs in the UK | 2000 - 2013 | 21 studies/ Sample size not reported | Computer simulation and other modelling methods have been used to analyse ED patient flow and resource capacity planning | Patient waiting times (i.e., time from arrival to discharge, admission or transfer), resources used, bed occupancy |
| **Advanced Nursing Role Interventions** | | | | | | |
| Carter, 2007, USA | Systematic review | To answer the question of whether hiring NPs for the ED can reduce wait time, improve patient satisfaction and provide care of reasonable quality and cost-effectiveness | NR (1979 – 2006) | 36 studies/ ED patients and staff, N= >52,000. Sample size not reported for all studies | NP role in ED | Cost-effectiveness, quality of care, patient satisfaction, wait times |
| Elder, 2015, Australia | Systematic review | To explore the literature regarding three key strategies designed to promote patient throughput in the ED | 1996 - 2013 | 21 studies/ 105,413 ED staff, patients. Sample size not reported for 1 study | Models of care: expanding nursing roles (e.g., ED NP, nurse- initiated x-ray, CIN), physician-assisted triage, medical assessment units | NP: wait times, unexpected representations, time to be seen by NP, ED LOS; Nurse initiated care: DNW rate, ED LOS, wait times, time to diagnosis, time to treatment, time to analgesia; Physician assisted triage: ED LOS, patient satisfaction, time to laboratory and diagnostic imaging, time to consultation, representation within 48 hours, DNW rates, time to review and disposition. Medical Assessment Unit: ED LOS, wait times for inpatient beds, number of patients waiting in ED for inpatient beds, admissions to medical wards, ED occupancy, overall inpatient days, cost saving, representation rate, readmission rate |
| Innes, 2015, Australia | Systematic review | To synthesize the findings of primary research examining healthcare roles introduced into ED waiting rooms | 2005 - 2013 | 6 studies/ >19,338,623 patients. Sample size not reported for all studies | Health professional roles in the waiting room | Waiting time, LWBS/ DNW, LOS, admissions, pathology testing, cannulation, ECG |
| Jennings, 2015, USA | Systematic review | To determine the impact of nurse practitioner services on cost, quality of care, satisfaction and waiting times in the emergency department for adult patients | 2006 - 2013 | 14 studies/ 36,621 patients presenting with soft tissue injury, patients presenting to ED, patients deemed low care, patients presenting with hand/wrist wounds/ fractures and removal of POP. Sample size not reported for all studies | NP in the ED: An independent nurse who is qualified to assess, diagnose and treat certain medical complaints. | Cost, quality of care, satisfaction, waiting times |
| Kleinpell, 2008, USA | Evidence-based review | To provide a summary of the results to date incorporating studies assessing the impact and outcomes of NPs and PAs in the ICU | 1990 - 2007 | 31 studies/ Nurse practitioners, physician assistants, physicians, residents and patients in acute and critical care settings. Sample size not reported for all studies | Advanced level practitioners including NPs, PAs, clinical nurse specialists, certified registered nurse anaesthetists, and certified nurse midwives | Patient satisfaction with ED care, number of medical histories, patients seeking unplanned follow-up advice, recovery times, levels of symptoms, time off work |
| Thamm, 2019, Australia | Systematic review | The primary objective of this review was to evaluate the effectiveness of nurse-led interventions generally on ED LOS | 1999 - 2013 | 6 studies/ 2,980 patients | Nurse-led interventions: APN roles (NP, ED NP) | LOS in ED, Adverse events (mortality, readmission, re-examination), patient satisfaction, cost effectiveness or utility |
| Williams, 2017, England | Literature review | To identify the evidence on the effects of advanced practitioners in emergency care | 2006 - 2016 | 4 studies/ 338,568 ED attendees, 190 ED staff | ANP, ACP, APN | Waiting time, time to intervention, assessment and treatment time, communication effectiveness, patient perception |
| Wylie, 2015, Australia | Systematic review | To identify current ED models of care and their impact on care quality, care effectiveness, and cost | Period of study not reported | 66 studies/ Sample size not reported | ED models of care: NP role, physician in triage, physician assistants and ED scribes, Care coordination teams, Fast-Track, rapid assessment units | NP role (wait time, LOS, patient satisfaction, errors, adverse events, representation rates, staff turnover), physician in triage (LOS, ambulance diversions, ), physician assistants and ED scribes (LOS), Care coordination teams (unnecessary social admission, re-presentation, staff satisfaction, retention rate), Fast-Track (wait time, LOS, costs, off stretcher times, ambulance diversion rates) |
| **Physiotherapy Interventions** | | | | | | |
| Anaf, 2007, Australia | Narrative review | To describe the role and responsibilities of an ED physiotherapist | 1996 -2006 | 9 studies/ 1903 patients. Unknown no. hospitals. Sample not specified for 3 studies. | Physiotherapy in the ED | Patient waiting times, patient satisfaction |
| Ferreira, 2019, Australia | Scoping review | To determine: 1. The roles undertaken by physiotherapists in the ED, their training levels, the profile of patients they treat and the types of care they provide; and 2. The effectiveness, efficiency, costs, and safety of care provided by physiotherapists working in the ED | 1996 - 2017 | 27 studies/ 165,244 patients, physiotherapists, physicians, residents | Physiotherapists working in an ED managing adult patients. | Wait time, treatment time, LOS, percent of patients discharged within emergency access benchmarks, quality of life, disability, days to return to work, proportion of patients returning to work within 30 days after ED presentation, time for patients managed by staff to return to daily activities, returned to leisure activities within 30 days, representing to the ED within 30 days of discharge, proportion of patients admitted from ED, treatment costs, cost per patient hour, direct costs, ED costs, hospital costs, total health costs, adverse events, adverse reactions to prescribed medications |
| Kilner, 2011, Australia | Systematic review | To determine what evidence there is that a physiotherapy service in the ED improves outcomes. | 1996 - 2008 | 11 studies/ ED staff and patients. Sample size not reported for all studies. | The addition of the physiotherapist to the ED multidisciplinary team. | Cost-effectiveness, hospital admissions, waiting time, referral rates, patient waiting times for physiotherapist, new patient non-attender rates, understanding & communication between physios & other ED staff, quality of life, health status, pain, patient satisfaction, time to usual activity |
| **General Practitioner Interventions** | | | | | | |
| Gonçalves-Bradley, 2018, England | Systematic review | To assess the effects of locating primary care professionals in hospital EDs to provide care for patients with non-urgent health problems, compared with care provided by regularly scheduled emergency physicians | 1995 - 2015 | 4 studies/ 11,463 patients, 16 GPs, nine emergency NPs, and 69 EPs (42 senior house officers, 25 registrars, and two consultants) | Primary care services: • within the ED, whereby patients enter the ED and are triaged into separate streams (broadly speaking urgent versus non-urgent); the non-urgent stream is staffed by primary care practitioners; • alongside the ED, whereby primary care is available onsite, next to the ED, and patients either self-select or are redirected from the ED towards the primary care service | Time from arrival to clinical assessment and treatment, ED LOS (from time of triage/registration to time of admission or discharge), diagnostic tests (overall number, cost), counselling, prescriptions, procedures, consultations or referrals to hospital-based specialists, arrangement of follow-up care, subsequent utilisation of primary care/re-attendance to the ED, patient education for self-management or appropriate service use, cost comparison (diagnostic tests investigations; treatment; referrals). mortality; self-reported health status; adverse events (return visits to the ED or readmissions) |
| Ramlakhan, 2016, England | Narrative literature review | To analyse the available literature in the context of the impact of GP delivered, hospital-based (adjacent or within the ED) unscheduled care services on process outcomes, cost-effectiveness and patient satisfaction | 1995 - 2014 | 20 studies/ 7,131 patients presenting to ED with non-urgent problems. Sample size not reported for all studies. | Models of hospital-based unscheduled care services that primarily use a workforce consisting of general practitioners or other primary care clinicians | Impact on attendances, process time measures, LOS, waiting time, treatment time, resource utilisation, radiography, laboratory use, medication, follow-up rates, admission, referral, reattendance/ re-consultation, cost effectiveness, patient satisfaction |
| **Scribe and Physician Assistant Interventions** | | | | | | |
| Cabilan, 2015, Australia | Review | To briefly overview the function of scribes and their potential contribution to Australian EDs | (2010 – 2014) | 7 studies/ Number of participants not reported | Medical scribes in ED | Patients per hour, time to clinician, time to disposition, ED LOS; job satisfaction, patient satisfaction, LWBS, cost per consultation |
| DeFreitas, 2018, England | Umbrella review | To summarise the evidence from systematic reviews on the interventions that improve patient flow in EDs | Reviews: 2006 - 2016 | 13 studies/ 2,225,339 ED staff, patients | Senior doctor triage, diagnostic services, assessment/short stay units, nurse directed interventions, administrative/organisational- e.g., scribes, and computerised provider order entry | Senior doctor triage (ED LOS, waiting time),diagnostic services, assessment/short stay units (ED LOS, Waiting time, physician initial assessment time), nurse directed interventions (ED LOS, patient off stretcher times), administrative/ organisational- e.g., scribes, (ED LOS, waiting times, door to room, room to doctor, time to disposition, patients per hour), and computerised provider order entry (ED LOS) |
| Doan, 2011, Canada | Systematic review | To assess the role of PAs in the ED, their impact on ED efficiency and on patient satisfaction | 1972 – 2009 | 66 studies/ Patients, PAs, ED Physicians, Sample size not reported for all studies | PAs in ED | Rate of investigations ordered, procedure performance, patient outcomes, documentation completeness, wait time, LOS, time to see a physician, assessment time, cost per visit. Physician attitudes, patient satisfaction |
| Heaton, 2016, USA | Systematic review and meta-analysis | To evaluate scribe effect on patient throughput, revenue, and patient and provider satisfaction. | 2004 - 2015 | 17 studies/ 231,129 patients of all ages seen in all clinical settings including ED, outpatient, and inpatient areas. Sample size not reported for all studies | Medical Scribes/ scribe services in ED: Non-medical persons whose role is to assist clinicians with non-clinical aspects of patient care such as documentation of patient notes and retrieval of investigations | Door to room, door to provider, room to provider, provider to disposition, door to disposition, LOS, patients per hour, billing (work relative value units), and patient and provider satisfaction |
| Wylie, 2015, Australia | Systematic review | To identify current ED models of care and their impact on care quality, care effectiveness, and cost | Period of study not reported | 66 studies/ Sample size not reported | ED models of care: NP role, physician in triage, physician assistants and ED scribes, Care coordination teams, Fast-Track, rapid assessment units | NP role (wait time, LOS, patient satisfaction, errors, adverse events, representation rates, staff turnover), physician in triage (LOS, ambulance diversions, ), physician assistants and ED scribes (LOS), Care coordination teams (unnecessary social admission, re-presentation, staff satisfaction, retention rate), Fast-Track (wait time, LOS, costs, off stretcher times, ambulance diversion rates) |
| **Pharmacy Interventions** | | | | | | |
| Cohen, 2009, USA | Systematic review | To ascertain the scope of involvement of clinical pharmacists in the ED; summarize economic, humanistic, and clinical outcomes data; describe current limitations of these data; and identify areas for future research | 1977 – 2008 | 16 studies/ 114 ED physicians, nurses. Sample size not reported for 14 studies | Clinical pharmacist in ED. For example, therapeutic consultation, dose and frequency adjustments, formulary interchange | Medication errors, cost-saving, cost avoidance, readmission rate, documentation completeness, acceptance ate/ staff perceptions/ staff attitudes |
| Hammond, 2019, USA | Scoping review | To identify, aggregate, and qualitatively describe the highest quality evidence for cost avoidance generated by clinical pharmacists on interventions performed in an ICU or ED | Period of study was not reported | 164 studies/ Sample size was not reported for all studies | Intervention that could be performed by a pharmacist in the ED and has some manner of Cost Avoidance (e.g., prevention of an adverse drug event) | Adverse drug event, minor adverse drug event, medication reconciliation, laboratory monitoring/ testing, resource utilization, inappropriate screening, guideline compliance, antimicrobial stewardship, cost avoidance |
| **Mental Health Services Interventions** | | | | | | |
| Callaghan, 2003, England | Systematic review | To review empirical research on the structure, process and outcome of liaison mental health services using systematic review methods | 1977 - 2001 | 48 studies/ 533 ED, ward, other healthcare staff, 1254 ED, ward, other patients/users. Sample size not reported for all studies | Liaison mental health services in the ED | Onward referral to specialist services, brief counselling/ psychotherapy, outpatient follow-up, prescribed medication, admitted to inpatient care, advice on health and social care issues |
| Evans, 2019, England | Systematic review | To evaluate the current evidence for what impact different Liaison Psychiatry services are having on ED | 2004 - 2018 | 17 studies/ Sample sizes ranged from 100 patients to 2715. Sample size not reported for each study | Liaison Psychiatry is a sub-specialty of multidisciplinary professionals. There are different models: ED boarding with psychiatry, area in ED with specialist psychiatric staff, liaison psychiatric services, psychiatric emergency service | LOS, time to assessment/ disposition |
| **Professional Development Interventions** | | | | | | |
| Boudreaux, 2004, USA | Systematic review | To answer two questions: 1) what are the strongest predictors of ED patient satisfaction? and 2) what changes can be made to improve ED patient satisfaction? | 1993 - 2002 | 50 studies/ 31,920 patients. Sample not specified for 37 studies | Patient satisfaction interventions: feedback of QI data and setting of benchmarks, videotape providing information about ED, MD/ RN patient care teams, information hand out, customer service training, 10 week medical Spanish course, chest pain observation unit, asthma observation unit, MD business cards, TVs in rooms, process redesign | Patient satisfaction with ED care |
| Boudreaux, 2006, USA | Critical review | To determine if Performance Improvement methods, in general, can be used to improve ED patient satisfaction, and which specific process changes or interventions show the most promise, either by way of head-to-head comparisons, larger effect sizes, or replication across different settings. | 1996 - 2004 | 19 studies/ 9,017 ED patients | Performance Improvement interventions: change to clinical practice guidelines, bedside registration, pathology protocols, patient assignment processes, customer service training, discharge planning, referral processes, ED Observation Units | Patient reported problems or patient complaints, patient satisfaction, patient waiting time satisfaction, Picker Institute Survey, patient rated nursing/doctor physician bedside manner/ technical skill |
| Lavoie, 2009, Canada | Systematic review | To determine what is currently known about outcome feedback in emergency medicine, including its incidence, impact and modifiers | 1992 - 2005 | 7 studies/ EM residents, EM physicians. Sample size not reported for all studies | Patient outcome feedback | Adverse events, perception of feedback |
| Lorenzetti, 2018, Canada | Systematic review | To conduct a systematic review of the effectiveness of interventions to improve the quality of ED physician documentation in emergency settings | 1989 - 2014 | 19 studies/ 10,801 ED physicians | Interventions to improve physician documentation: audit/feedback, dictation, education, facilitation, reminders, structured paper templates | Quality of physician feedback |
| McCaughey, 2015, USA | Systematic review | To review and synthesize the current academic literature regarding capacity management that focuses on the ED, identify strengths and weaknesses of the approaches in the literature, and provide practical recommendations for hospital administrators | 2001 - 2012 | 23 studies/ Sample size not reported | Bed management scheduling, triage protocols, cross-training nurses, outsourcing environmental services, fast-track, treatment protocols, | ED indicators (volume, LOS, Admission rate), Lost business (LWBS, transfer centre declines), Staff efficiency (Environmental services turn-around time, transport), time stamps (door-to-provider time, turn-around time, order-to-results time), patient satisfaction |
| Rogers, 2015, USA | Systematic review | To assess the effect of audit and feedback on emergency physician performance and identify features critical to success | 1995 - 2014 | 24 studies/ ED physicians, interns, residents, health personnel. Sample size not reported for all studies | Audit and feedback interventions for physicians | Physician behaviour, treatment effect based on provider performance |
| Sampson, 2014, England | Systematic review | To identify interventions that could improve pain management in the ED. | 1995 - 2012 | 42 studies/ 68,727 patients presenting to ED | Interventions to change professional behaviours around pain management in ED | Proportion of patients receiving analgesia, time to analgesia, change in pain score, proportion of patients receiving adequate analgesia, documentation of pain score, reassessment of pain, repeat dosing of analgesia, patient satisfaction |
| Seo, 2019, South Korea | Systematic review | To examine the hand hygiene compliance rate, factors affecting the hand hygiene compliance rate, and intervention strategies to improve hand hygiene compliance in EDs. | 1994 - 2017 | 24 studies/ Nurses, physicians, residents, healthcare assistants, and therapists. Sample size not reported | Interventions to improve hand hygiene compliance in the ED | Hand hygiene compliance rates, ED workload, attitudes |
| Williams, 2019, Australia | Integrative literature review | To explore systems level change in the ED for improved paediatric pain management. | 2001 - 2016 | 20 studies/ 6,641 patients. sample sizes ranged from 52 to 1,200, with three studies including less than 100 participants. | Evidence-based protocols or policy, nurse-initiated analgesia, clinical education, family/ patient involvement | Nurse-initiated analgesia (time to analgesia, provision of analgesia, parental satisfaction), clinical education (provision of analgesia, documentation of pain scores), family/ patient involvement (pain score, pain beliefs, time to analgesia) |
| **Reviews addressing the role of patients in ED performance** | | | | | | |
| Flynn, 2012, England | Systematic review | To conduct a systematic review to evaluate the approaches, methods, and tools used to engage patients or their surrogates in shared decision-making in the ED | 1997 - 2012 | 5 studies/ 1,009 patients and surrogates | Patient involvement and engagement in decision-making in the ED setting via the use of decision support interventions. Patient decisions aids and structured decision support interventions are the most common tools used to support shared decision making in clinical encounters | Preference and decision-making, readmission, patient satisfaction, LOS, patient knowledge & involvement in decision-making |
| Williams, 2019, Australia | Integrative literature review | To explore systems level change in the emergency department  for improved paediatric pain management. | 2001 - 2016 | 20 studies/ 6,641 patients. sample sizes ranged from 52 to 1,200, with three studies including less than 100 participants. | Evidence-based protocols or policy, nurse-initiated analgesia, clinical education, family/ patient involvement | Nurse-initiated analgesia (time to analgesia, provision of analgesia, parental satisfaction), clinical education (provision of analgesia, documentation of pain scores), family/ patient involvement (pain score, pain beliefs, time to analgesia) |
| ACP, advanced clinical practitioner; ANP, advanced nurse practitioner; APN, Advanced Practice Nurse; ASA, acetylsalicylic acid; CIN, clinical initiatives nurse; CT, computerised tomography; DeMIST, demographics, mechanism of injury or illness, injuries, signs, treatment given; DNA, did not arrive; DNW, did not wait; ECG, electrocardiogram; ED, Emergency Department; ED LOS, Emergency Department length of stay; ED NP, Emergency Department nurse practitioner; EM, emergency medicine; EMS, emergency medical services; EMT, emergency medical technician; ENP, emergency nurse practitioner; GP, general practitioner; ICU, Intensive Care Unit; IMIST-AMBO, identification, mechanism/ medical complaint, injuries/information, signs, treatment and trends – allergies, medication, background history, other information; LOS, length of stay; LWBS, left without being seen; MD, medical doctor/ physician; MI, myocardial infarction; N, number; NEAT, Australian national emergency access target; No., number; NP, nurse practitioner; NR, not reported; PA, physician assistant; POP, plaster of paris; QI, quality improvement; QOL, quality of life; RN, registered nurse; TV, television | | | | | | |
