# Supplementary material for: Strategies to measure and improve emergency department performance: a scoping review
Source: Scand J Trauma Resusc Emerg Med. 2020 Jun 15;28:55. doi: 10.1186/s13049-020-00749-2 (PMC7296671; doi:10.1186/s13049-020-00749-2)
Supplement: Supplementary file 4 — Additional file 4: Table 3. The evidence contribution to the review questions of the included reviews. [file 13049_2020_749_MOESM4_ESM.docx]

**Table 3. The evidence contribution to the review questions of the included reviews.**

|  | **Review Question** | | | |
| --- | --- | --- | --- | --- |
| **Author, Year** | **1.How is ED performance measured?** | **2.What are the interventions used to improve ED performance?** | **3.What is the role(s) of patients in improving ED performance?** | **4.What are the outcomes attributed to interventions used to improve ED performance** |
| Abdulwahid, 2016 |  | YES |  | YES |
| Alimenti, 2019 |  | YES |  | YES |
| Anaf, 2007 |  | YES |  | YES |
| Bennett, 2017 |  | YES |  | YES |
| Bingisser, 2012 |  | YES |  | YES |
| Boudreaux, 2004 |  | YES |  | YES |
| Boudreaux, 2006 |  | YES |  | YES |
| Bowden, 2017 |  | YES |  | YES |
| Bucci, 2016 |  | YES |  | YES |
| Bullard, 2012 |  | YES |  | YES |
| Cabilan, 2015 |  | YES |  | YES |
| Cabilan, 2017 |  | YES |  | YES |
| Callaghan, 2003 |  | YES |  | YES |
| Carter, 2007 |  | YES |  | YES |
| Chhabra, 2019 |  | YES |  | YES |
| Cohen, 2009 |  | YES |  | YES |
| Considine, 2019 |  | YES |  | YES |
| Curran, 2019 |  | YES |  | YES |
| Dawson, 2013 |  | YES |  | YES |
| De Freitas, 2018 |  | YES |  | YES |
| Deblois, 2018 |  | YES |  | YES |
| Desai, 2018 |  | YES |  | YES |
| Dexheimer, 2015 |  | YES |  | YES |
| Doan, 2011 |  | YES |  | YES |
| Doan, 2014 |  | YES |  | YES |
| Elder, 2015 |  | YES |  | YES |
| Evans, 2019 |  | YES |  | YES |
| Ferreira, 2019 |  | YES |  | YES |
| Flynn, 2012 |  |  | YES | YES |
| Flynn, 2016 |  | YES |  | YES |
| Galipeau, 2015 |  | YES |  | YES |
| Georgiou, 2013 |  | YES |  | YES |
| Gonçalves-Bradley, 2018 |  | YES |  | YES |
| Goodacre, 2000 |  | YES |  | YES |
| Hammond, 2019 |  | YES |  | YES |
| Harding, 2011 |  | YES |  | YES |
| Heaton, 2016 |  | YES |  | YES |
| Hersh, 2015 |  | YES |  | YES |
| Holden, 2011 |  | YES |  | YES |
| Hoot, 2008 |  | YES |  | YES |
| Hughes, 2019 |  | YES |  | YES |
| Innes, 2015 |  | YES |  | YES |
| Isfahani, 2019 |  | YES |  | YES |
| Jennings, 2015 |  | YES |  | YES |
| Jones, 2010 |  | YES |  | YES |
| Juillard, 2009 |  | YES |  | YES |
| Kelton, 2018 |  | YES |  | YES |
| Kilner, 2011 |  | YES |  | YES |
| Kirkland, 2019 |  | YES |  | YES |
| Kleinpell, 2008 |  | YES |  | YES |
| Konnyu, 2012 |  | YES |  | YES |
| Kumar, 2013 |  | YES |  | YES |
| Lavoie, 2009 |  | YES |  | YES |
| Lorenzetti, 2018 |  | YES |  | YES |
| Madsen, 2015 | YES |  |  |  |
| McCaughey, 2015 |  | YES |  | YES |
| Mieiro, 2019 |  | YES |  | YES |
| Ming, 2016 |  | YES |  | YES |
| Mohiuddin, 2017 |  | YES |  | YES |
| Morley, 2018 |  | YES |  | YES |
| Oredsson, 2011 |  | YES |  | YES |
| Ramlakhan, 2016 |  | YES |  | YES |
| Reay, 2019 |  | YES |  | YES |
| Rehman, 2016 |  | YES |  | YES |
| Robinson, 2013 |  | YES |  | YES |
| Rogers, 2015 |  | YES |  | YES |
| Rowe, Guo, 2011 |  | YES |  | YES |
| Rowe, Villa-Roel, 2011 |  | YES |  | YES |
| Sampson, 2014 |  | YES |  | YES |
| Seo, 2019 |  | YES |  | YES |
| Shankar, 2014 |  | YES |  | YES |
| Sørup, 2013 | YES |  |  |  |
| Stang, 2015 | YES |  |  |  |
| Thamm, 2019 |  | YES |  | YES |
| Williams, 2017 |  | YES |  | YES |
| Williams, 2019 |  | YES | YES | YES |
| Wylie, 2015 |  | YES |  | YES |

‘Yes’ means that the included review contributes evidence to the review question.
