# Supplementary material for: Strategies to measure and improve emergency department performance: a scoping review
Source: Scand J Trauma Resusc Emerg Med. 2020 Jun 15;28:55. doi: 10.1186/s13049-020-00749-2 (PMC7296671; doi:10.1186/s13049-020-00749-2)
Supplement: Supplementary file 5 — Additional file 5: Table 4. The ROBIS Risk of Bias results for each domain and the overall risk of bias for the included reviews [file 13049_2020_749_MOESM5_ESM.doc]

**Table 4.** **The ROBIS Risk of Bias results for each domain and the overall risk of bias for the included reviews**

|  | **Phase 2** | | | | **Phase 3** |
| --- | --- | --- | --- | --- | --- |
| **Author, Year** | **1.       Study eligibility criteria** | **2. Identification and selection of studies** | **3. Data collection and study appraisal** | **4. Synthesis and findings** | **Overall risk of bias in the review** |
| **Abdulwahid, 2016** | LOW | UNCLEAR | LOW | LOW | LOW |
| **Alimenti, 2019** | HIGH | LOW | UNCLEAR | LOW | UNCLEAR |
| **Anaf, 2007** | LOW | LOW | LOW | LOW | LOW |
| **Bennett, 2017** | LOW | LOW | UNCLEAR | LOW | LOW |
| **Bingisser, 2012** | UNCLEAR | UNCLEAR | UNCLEAR | UNCLEAR | HIGH |
| **Boudreaux, 2004** | LOW | LOW | LOW | HIGH | HIGH |
| **Boudreaux, 2006** | LOW | LOW | UNCLEAR | UNCLEAR | UNCLEAR |
| **Bowden, 2017** | LOW | LOW | HIGH | LOW | HIGH |
| **Bucci, 2016** | LOW | LOW | UNCLEAR | UNCLEAR | UNCLEAR |
| **Bullard, 2012** | LOW | LOW | LOW | LOW | LOW |
| **Cabilan, 2015** | UNCLEAR | UNCELAR | UNCLEAR | UNCLEAR | HIGH |
| **Cabilan, 2017** | LOW | LOW | LOW | LOW | LOW |
| **Callaghan, 2003** | LOW | UNCLEAR | UNCLEAR | UNCLEAR | UNCLEAR |
| **Carter, 2007** | LOW | LOW | LOW | UNCLEAR | UNCLEAR |
| **Chhabra, 2019** | LOW | LOW | LOW | LOW | LOW |
| **Cohen, 2009** | UNCLEAR | HIGH | HIGH | HIGH | HIGH |
| **Considine, 2019** | LOW | LOW | LOW | LOW | LOW |
| **Curran, 2019** | LOW | LOW | LOW | LOW | LOW |
| **Dawson, 2013** | LOW | HIGH | LOW | UNCLEAR | HIGH |
| **De Freitas, 2018** | HIGH | LOW | LOW | LOW | HIGH |
| **Deblois, 2018** | HIGH | LOW | HIGH | LOW | HIGH |
| **Desai, 2018** | UNCLEAR | LOW | LOW | LOW | LOW |
| **Dexheimer, 2015** | LOW | LOW | UNCLEAR | UNCLEAR | HIGH |
| **Doan, 2011** | LOW | UNCLEAR | UNCLEAR | UNCLEAR | UNCLEAR |
| **Doan, 2014** | LOW | LOW | LOW | LOW | LOW |
| **Elder, 2015** | LOW | HIGH | LOW | LOW | LOW |
| **Evans, 2019** | LOW | LOW | LOW | LOW | LOW |
| **Ferreira, 2019** | LOW | LOW | HIGH | UNCLEAR | UNCLEAR |
| **Flynn, 2012** | LOW | LOW | LOW | LOW | LOW |
| **Flynn, 2016** | LOW | LOW | LOW | LOW | LOW |
| **Galipeau, 2015** | LOW | LOW | LOW | LOW | LOW |
| **Georgiou, 2013** | LOW | LOW | HIGH | LOW | HIGH |
| **Gonçalves-Bradley, 2018** | LOW | LOW | LOW | LOW | LOW |
| **Goodacre, 2000** | UNCLEAR | HIGH | HIGH | LOW | HIGH |
| **Hammond, 2019** | LOW | LOW | HIGH | LOW | HIGH |
| **Harding, 2011** | LOW | LOW | LOW | LOW | LOW |
| **Heaton, 2016** | LOW | LOW | LOW | LOW | LOW |
| **Hersh, 2015** | LOW | LOW | LOW | LOW | LOW |
| **Holden, 2011** | UNCLEAR | LOW | HIGH | UNCLEAR | HIGH |
| **Hoot, 2008** | UNCLEAR | HIGH | UNCLEAR | UNCLEAR | HIGH |
| **Hughes, 2019** | LOW | LOW | LOW | LOW | LOW |
| **Innes, 2015** | LOW | UNCLEAR | LOW | LOW | HIGH |
| **Isfahani, 2019** | LOW | UNCLEAR | UNCLEAR | HIGH | HIGH |
| **Jennings, 2015** | HIGH | HIGH | LOW | LOW | HIGH |
| **Jones, 2010** | LOW | LOW | UNLCEAR | UNCLEAR | UNCLEAR |
| **Juillard, 2009** | UNCLEAR | HIGH | HIGH | HIGH | HIGH |
| **Kelton, 2018** | HIGH | LOW | HIGH | UNCLEAR | HIGH |
| **Kilner, 2011** | LOW | LOW | HIGH | UNCLEAR | HIGH |
| **Kirkland, 2019** | LOW | LOW | LOW | LOW | LOW |
| **Kleinpell, 2008** | LOW | HIGH | HIGH | HIGH | HIGH |
| **Konnyu, 2012** | UNCLEAR | LOW | HIGH | LOW | HIGH |
| **Kumar, 2013** | LOW | UNCLEAR | LOW | UNCLEAR | UNCLEAR |
| **Lavoie, 2009** | LOW | LOW | HIGH | HIGH | HIGH |
| **Lorenzetti, 2018** | LOW | LOW | LOW | LOW | LOW |
| **Madsen, 2015** | LOW | LOW | UNCLEAR | UNCLEAR | UNCLEAR |
| **McCaughey, 2015** | LOW | HIGH | UNCLEAR | UNCLEAR | HIGH |
| **Mieiro, 2019** | LOW | LOW | UNCLEAR | LOW | UNCLEAR |
| **Ming, 2016** | LOW | UNCLEAR | LOW | LOW | UNCLEAR |
| **Mohiuddin, 2017** | LOW | LOW | HIGH | UNCLEAR | LOW |
| **Morley, 2018** | LOW | LOW | LOW | LOW | LOW |
| **Oredsson, 2011** | LOW | UNCLEAR | LOW | LOW | UNCLEAR |
| **Ramlakhan, 2016** | HIGH | LOW | HIGH | HIGH | HIGH |
| **Reay, 2019** | LOW | LOW | LOW | LOW | LOW |
| **Rehman, 2016** | LOW | UNCLEAR | LOW | UNCLEAR | HIGH |
| **Robinson, 2013** | HIGH | UNCLEAR | HIGH | LOW | HIGH |
| **Rogers, 2015** | LOW | LOW | LOW | LOW | LOW |
| **Rowe, Guo, 2011** | LOW | LOW | LOW | LOW | LOW |
| **Rowe, Villa-Roel, 2011** | LOW | LOW | LOW | LOW | LOW |
| **Sampson, 2014** | LOW | LOW | HIGH | LOW | HIGH |
| **Seo, 2019** | LOW | LOW | LOW | LOW | LOW |
| **Shankar, 2014** | LOW | HIGH | UNCLEAR | UNCLEAR | UNCLEAR |
| **Sørup, 2013** | LOW | LOW | HIGH | HIGH | HIGH |
| **Stang, 2015** | LOW | LOW | LOW | LOW | LOW |
| **Thamm, 2019** | LOW | LOW | LOW | LOW | LOW |
| **Williams, 2017** | LOW | UNCLEAR | HIGH | UNCLEAR | HIGH |
| **Williams, 2019** | LOW | UNCLEAR | UNCLEAR | LOW | UNCLEAR |
| **Wylie, 2015** | UNCLEAR | UNCLEAR | UNCLEAR | UNCLEAR | HIGH |

“LOW” means that the included review was rated as having a low risk of bias. “HIGH” means that the included review was rated as having a high risk of bias. “UNCLEAR” means the included review was rated as having insufficient information reported to make a judgement on risk of bias.
