# Supplementary material for: Strategies to measure and improve emergency department performance: a scoping review
Source: Scand J Trauma Resusc Emerg Med. 2020 Jun 15;28:55. doi: 10.1186/s13049-020-00749-2 (PMC7296671; doi:10.1186/s13049-020-00749-2)
Supplement: Supplementary file 6 — Additional file 6: Table 14. The performance measures reported by the included reviews for each intervention. [file 13049_2020_749_MOESM6_ESM.docx]

**Table 14. The performance measures reported by the included reviews for each intervention**

| **Intervention** | **Time**  **No.- measure** | **Proportion No.- measure** | **Process**  **No.- measure** | **Cost**  **No.- measure** | **Clinical**  **No.- measure** |
| --- | --- | --- | --- | --- | --- |
| **Practices and Processes** | | | | | |
| **Triage Interventions** | | | | | |
| Senior Doctor Triage/ Team triage | 10- ED LOS (15–23), waiting time (15–17,22,23), transit time (15), triage time (15), time to be seen (physician/ provider) (20), door to doctor time (20), time to patient disposition decision (20), physician initial assessment time (21), time to consultation (18), time to review and disposition (18) | 2- Ambulance diversion/ bypass (hours) (19,20), number on waiting list (15) | 8- LWBS/ DNW (15,16,18,20–22),left without treatment complete (16), patient satisfaction (18,19,25), patient disappearance (23), hours of access block/ boarding hours (21), adherence to guidelines (21), leaving against medical advice (21) | 2- Salary, staff turnover (19) | 6- Patient reattendance (19,23), unscheduled returns (20), representation with in 48hours (18), adverse events (15,19), clinical errors (19), mortality (20,23) |
| Dedicated Triage Resources | 1- Door-to-ECG time (24) | - NA | - NA | - NA | - NA |
| Triage Education | 1- Door-to-ECG time (24) | - NA | - NA | - NA | - NA |
| Triage Systems | 4- ED LOS, waiting time, transit time, triage time (15) | 1- Number on the waiting list (15) | 3- LWBS/ DNW, did not arrive (15) | - NA | 1- Adverse events (15) |
| Triage Protocols | 2- LOS after provider assessment, LOS in relation to acuity level (25) | 3- Rates of unnecessary radiograph request, rate of radiographs requested after assessment, rate of positive abnormalities on radiographs (25) | 1- Patient satisfaction (25) | - NA | - NA |
| Nurse-led Triage | - NA | - NA | 1- Patient satisfaction (27) | - NA | - NA |
| **Care Transitions (Handover processes) Interventions** | | | | | |
| Handover Tools | 2- ED LOS, Wait time (31) | 3- Types and characteristics of handover (28), information recall (28), Physician recall (30) | 8- Perceptions of patient safety (29), perception of utility (28), quality of handover (28), adherence to guidelines (30,31), documentation quality (30), LWBS (31), communication between EMS providers and ED nurses (31), effectiveness of the transition (31) | - NA | - NA |
| Bedside Registration | 9- ED LOS, ambulance diversion time (hours), ambulance transfer of care time, time from triage to room, time from room to disposition, time to diagnostic test, time to treatment, mean transit times within the ED, time to first ED clinician review (20) | 6- Proportion of patients meeting NEAT, ED Occupancy/ rate, staff perception, proportion of patients leaving ED within 4 hours, Inpatient LOS, proportion of admissions meeting NEAT (20) | 7- LWBS, NEAT compliance, access block (20), patient wait time satisfaction, patient rated nursing/doctor physician bedside manner/ technical skill, patient reported problems or patient complaints, patient satisfaction (32) | - NA | 4- Mortality, reattendances/ representation, readmission, 48 hour representation (20) |
| Discharge Planning | - NA | - NA | 4- Patient wait time satisfaction, patient rated nursing/doctor physician bedside manner/ technical skill, patient reported problems or patient complaints, patient satisfaction (32) | - NA | - NA |
| Discharge Communication | - NA | 3- Parent recall of discharge information, knowledge and comprehension, discharge advice (33) | 2- Adherence to guidelines, provider behaviour (33) | - NA | 3- Unnecessary ED return visits, knowledge acquisition, asthma management (33) |
| Discharge Protocols | 3- ED LOS, wait time (31), door-to-CT scan time (30) | 3- Types and characteristics of handover, information recall (28), physician recall (30) | 7- Perception of utility, quality of handover (28), documentation quality (30), adherence to guidelines (30,31), LWBS, communication between EMS providers and ED nurses, effectiveness of the transition (31) | - NA | - NA |
| Discharge Clinical Roles | 2- ED LOS (31), wait time (31,34) | - NA | 5- Adherence to guidelines, LWBS, communication between EMS providers and ED nurses, effectiveness of the transition (31), patient satisfaction (34) | - NA | 1- Unscheduled admissions within 14 days of discharge (34) |
| **Process Re-design Interventions** | | | | | |
| Guidelines and Protocols | 3- Time to diagnostic test, average length of hospital stay, turnaround time (36) | 3- Provision of analgesia (43), infection rate, patient call backs (36) | 4- Patient satisfaction, patient complaints, left against medical advice (36), parental satisfaction (43) | 1- Cost saving (36) | 5- Medication errors (35), mortality, error rate (36), pain score, pain beliefs (43) |
| Patient Assignment and Referral Processes | 1- ED LOS (2) | 5- Ambulance arrivals, need for diversion (2), ED attendance (37), ED utilisation, inpatient admission rate (39) | 1- Patient experience (38) | 2- Cost effectiveness (37), cost (39) | 5- Re-attendances/ re-presentation (37,38), quality of life (38), hospitalization at or after the initial ED index visit (37,38), use of other healthcare services (37), psychosocial outcomes (39) |
| Organisational Processes | 5- Time to diagnostic test, average length of hospital stay, turnaround time (36), time to first ED clinician review, surgery wait times (41) | 7- Infection rate, patient call backs (36), tests per patient, admissions, admissions <24hr, presentations, attitudes/beliefs about 4hr target (41) | 5- Patient Satisfaction (40), DNW, treated <1hr (41), patient complaints, left against medical advice (36) | 1- Cost saving (36) | 3- Mortality (36,41), error rate (36), return <7 days (41) |
| Nurse-Initiated Care Processes | 11- ED LOS (17), time from triage to ED discharge (44), wait time (18,22), time from triage to medical assessment (44), time to treatment (18,42), time to first ED clinician review (45), patient off stretcher time (17), time to diagnosis (18), time to analgesia (18,42,43), doctor wait time (42), time to x-ray (44) | 3- Access to analgesia (42), proportion of radiographs ordered(45), provision of analgesia (43) | 3- Patient Satisfaction (42,44), DNW (18), accuracy of x-ray request (44) | - NA | 9- Clinical improvement, clinically relevant pain relief, nausea and vomiting, medication errors, perception of tremors and palpitation, ED representations, deviation of vital signs (42), pain score, pain beliefs (43) |
| Clinical Decision Supports | - NA | 4- Imaging use/ number of scans ordered (46,47), diagnostic yield (47), change in imaging ordering (48), admissions (46) | 1- Adherence to guidelines (47) | - NA | - NA |
| Lean Management/ Thinking | 16- LOS, waiting time (49–51), median exam room time (49), time to physician assessment, login to disposition time, login to triage time, lead time, triage waiting time, waiting time for consultation, admission waiting time, registration to physician time, time spent in examining area by patient, time to ECG, time to physician assessment, time to ASA administration, door to doctor time (51) | 8- Patient volume, number of admitted patients, number of patients discharged, number of patients seen and discharged within 4 hours (49), proportion of cases with 12 lead ECG completed within 10 minutes of triage, physician assessment initiated within 60 minutes, number of searches of supplies by nurses, number of patients discharges in <1hr (51) | 2- Patient satisfaction, LWBS (49–51) | 2- Direct expense (49), cost per patient (51) | - NA |
| **Point-of-Care Testing Interventions** | | | | | |
| Point of Care Testing | 3- ED LOS (17,22,52,53), waiting time (17), response time (22) | 7- Reliability of point of care testing for cardiac troponin (52), patients per hour, antimicrobial prescription rate in the ED, rate of ancillary tests, rate of physician visit within 2 weeks, hospital admission rate, acceptability of nasal specimen collection sampling for rapid viral testing (53) | 3- Management of patients (52), LWBS, DNW (20) | 1- ‘cost’ (52) | 1- Impact of point of care testing for cardiac troponin on patient outcome/safety (52) |
| **Observation Unit Interventions** | | | | | |
| Condition Specific Observation Unit | - NA | - NA | 2- Patient satisfaction (32,40), LWBS (54) | - NA | - NA |
| Rapid Assessment Zone/ Pod | 4- Wait time, off stretcher time (19), ED LOS, Physician initial assessment time (56) | 1- Ambulance diversion (19) | - NA | 1- Cost saving (56), costs (19) | 3- Re-presentation rate, mortality, adverse events (54) |
| Medical Assessment Unit | 1- ED LOS (2,17,18) | 5- number of patients waiting for inpatient beds (2,18), admissions to medical wards, ED occupancy, overall inpatient days (18), ambulance diversion (2) | 1- LWBS (2) | 1- Cost saving (18) | 2- Re-presentation rate, re-admission rate (18) |
| Short Stay Unit | 2- ED LOS (17,18,55,57), Wait time (17,18) | 4- Number of patients waiting for inpatient beds, admissions to medical wards, ED occupancy, overall inpatient days (18) | 12- Patient satisfaction, communication, special needs preferences, perceived length of stay, discharge preparation, pain management, family involvement, patient education, physical comfort, emotional support, financial information, missed pathology (57) | 2- Cost effectiveness (57) Cost saving (18) | 2- Re-presentation rate (18), re-admission rate (18,55) |
| ED Managed Acute Care Unit | 2- Wait time, ED LOS (20) | - NA | - NA | - NA | - NA |
| Quick Diagnosis Units | 3- Wait time, ED LOS, off stretcher time (19) | 1- Ambulance diversion (19) | - NA | 1- Costs (19) | - NA |
| **Technology Interventions** | | | | | |
| Computerised Clinical Support Systems | 9- ED LOS (46,59), time to measurement (various), timeliness of tests/ imaging (46), time spent on the computer, time spent using paper, or time spent on patient care, turnaround time, time between 2 points (e.g., interval between a patient arriving in the ED and a test being ordered), time until order renewal (59) | 4- Admission rate, number of scans ordered (46), number of tests ordered, prescription rate (59) | 5- Quality of care (46), use of decision support system, compliance or non-compliance with recommendations/ guidelines, potential adverse events(59), specimen processing errors (59) | 1- Cost of care (46) | 3- Medication error rate, case fatality rate, time in restraints (59) |
| Mobile Devices | 5- Efficiency of the software, efficiency of the provider, access time, user perceptions of speed, time spent accessing information (58) | - NA | 1- Guideline accuracy (58) | - NA | 1- Error rates (58) |
| Telecommunication Technology | - NA | - NA | 1- Patient satisfaction (60) | - NA | 2- Return <3 days, need for further treatment (60) |
| Computer Simulation | 1- Patient wait times (i.e., time from arrival to discharge, admission or transfer) (61) | 2- Resource use, bed occupancy (61) | - NA | - NA | - NA |
| eHealth Records Access | 1- ED LOS (63) | 3- Admission rate, lab/ radiology testing rate, frequent users (63) | 5- Clinician utilisation rate (62), quality of care, referrals and consultations, public health reporting, patient/ clinician perception (63) | 1- Cost (63) | 1- Re-admissions (63) |
| **Team Composition** | | | | | |
| **Advanced Nursing Role Interventions** | | | | | |
| Nurse Practitioner | 2- ED LOS (18,19,67), Waiting time (18,19,64,65,68,69) | 5- Quality of care, patient satisfaction (64) staff satisfaction, staff turnover, retention rate (19) | 4- Patient satisfaction (19,65–67), quality of care (65), communication effectiveness, patient perception (68) | 2- Cost (65), Cost effectiveness (64,67) | 7- Unexpected re-presentation, adverse events (19,67), mortality, re-admission, re-examination (67), errors, unnecessary social admission (19) |
| Clinical Nurse Specialist | - NA | - NA | - NA | - NA | - NA |
| Certified Registered Nurse Anaesthetists | - NA | - NA | - NA | - NA | - NA |
| Clinical Initiatives Nurse | 3- ED LOS, wait time (18,69), time to be seen by NP (18) | 4- Admissions, pathology testing/ resource use, cannulation, ECG (69) | 2- Patient satisfaction (18), LWBS/ DNW (69) | - NA | 1- Unexpected representation (69) |
| **Physiotherapy Interventions** | | | | | |
| Physiotherapy Services | 3- ED LOS, assessment and treatment time (71), waiting times (70–72) | 7- Percent of patients discharged within emergency access benchmarks, proportion of patients admitted from ED (71), admissions, referral rates, new patient non-attender rates, understanding and communication between physios & other ED staff (72) | 2- Patient satisfaction (70,72), quality of life (71,72) | 7- Treatment costs, cost per patient hour, direct costs, ED costs, hospital costs, total health costs (71), Cost effectiveness (72) | 10- Proportion of patients returning to work within 30 days after ED presentation, disability, days to return to work, returned to leisure activities within 30 days, representing to the ED within 30 days of discharge, adverse events, adverse reactions to prescribed medications (71), health status, pain (72), time for patients to return to daily activities (71,72) |
| **General Practitioner Interventions** | | | | | |
| General Practitioners | 3- ED LOS, assessment and treatment time (73,74), waiting times (74) | 7- admissions (73,74), number of diagnostic tests, counselling, prescriptions, procedures (73), follow-up rate, pathology testing/ resource use (74) | 5- Consultations or referrals to hospital-based specialists, arrangement of follow-up care, patient education for self-management or appropriate service use (73), referral, patient satisfaction (74) | 3- Cost of diagnostic tests, cost comparison of: diagnostic tests/investigations; treatment; referrals (73), Cost effectiveness (74) | 5- Subsequent utilisation of primary care/re-attendance to the ED, mortality, self-reported health status, adverse events (return visits to the ED or readmissions) (73), re-presentation (74) |
| **Scribe and Physician Assistant Interventions** | | | | | |
| Scribes | 9- ED LOS (17,75,77), waiting times (17,19), door to room (17,77), room to doctor (17,77), time to disposition (17,75), time to clinician (75), door to provider, provider to disposition, door to disposition (77) | 5- Patients per hour (17,75,77), job satisfaction (75), staff satisfaction (19,77), staff turnover (19), retention rate (19) | 2- Patient satisfaction (19,75,77), LWBS/ DNW (75) | 2- Billing: work relative value units (77), cost per consultation (75) | 4- Errors, adverse events, re-presentation, unnecessary social admission (19) |
| Physician Assistants | 6- ED LOS, waiting times, time to clinician (76), door to room, room to doctor, time to disposition (17) | 7- Patients per hour (17), staff turnover, retention rate, staff satisfaction (19), rate of investigations ordered, procedure performance, physician attitudes (76) | 2- Patient satisfaction (19,76), documentation completeness (76) | 1- Cost per visit (76) | 5- Patient outcomes (76), errors, adverse events, re-presentation, unnecessary social admission (19) |
| **Pharmacy Interventions** | | | | | |
| Pharmacy | - NA | 3- Pathology testing/ resource use, inappropriate screening (79), staff acceptance/ perception/ attitude (78) | 4- Guideline compliance, antimicrobial stewardship, medication reconciliation (79), documentation completeness (78) | 2- Cost avoidance (78,79), cost saving (78) | 3- Medication errors, readmission (78), adverse events (79) |
| **Mental Health Services Interventions** | | | | | |
| Liaison Mental Health Services | 3- ED LOS, time to disposition, assessment and treatment time (81) | 6- Admissions, prescriptions, onward referral to specialist services, brief counselling/ psychotherapy, outpatient follow-up, advice on health and social care issues (80). | - NA | - NA | - NA |
| Co-located Psychiatry Liaison Personnel/ Spaces | 3- ED LOS, time to disposition, assessment and treatment time (81) | - NA | - NA | - NA | - NA |
| Psychiatry Specialist Services | 3- ED LOS, time to disposition, assessment and treatment time (81) | - NA | - NA | - NA | - NA |
| **Professional Development Interventions** | | | | | |
| Professional Development | 1- Time to analgesia (86) | 4- Provision of analgesia (43), proportion of patients receiving analgesia, proportion of patients receiving adequate analgesia (86), workload attitudes (85) | 10- Documentation of pain scores (43,86), Patient satisfaction (32,40,86), perception of feedback (82), physician behaviour, provider performance, quality of physician feedback (83), change in pain score, reassessment of pain, repeat dosing of analgesia (86), hand hygiene compliance rate (85) | - NA | 1- Adverse events (82) |
| ASA, acetylsalicylic acid; CT, computerised tomography; DNW, did not wait; ED, Emergency Department; ED LOS, Emergency Department length of stay; ECG, electrocardiogram; EMS, emergency medical services; LWBS, left without being seen; LOS, length of stay; NA, not applicable; NEAT, Australian national emergency access target | | | | | |
